# Meta-analysis of up to 622,409 individuals identifies 40 novel smoking behaviour associated genetic loci

**DOI:** 10.1038/s41380-018-0313-0

**Published:** 2019-01-07

**Authors:** A. Mesut Erzurumluoglu, Mengzhen Liu, Victoria E. Jackson, Daniel R. Barnes, Gargi Datta, Carl A. Melbourne, Robin Young, Chiara Batini, Praveen Surendran, Tao Jiang, Sheikh Daud Adnan, Saima Afaq, Arpana Agrawal, Elisabeth Altmaier, Antonis C. Antoniou, Folkert W. Asselbergs, Clemens Baumbach, Laura Bierut, Sarah Bertelsen, Michael Boehnke, Michiel L. Bots, David M Brazel, John C. Chambers, Jenny Chang-Claude, Chu Chen, Janie Corley, Yi-Ling Chou, Sean P. David, Rudolf A. de Boer, Christiaan A. de Leeuw, Joe G. Dennis, Anna F. Dominiczak, Alison M. Dunning, Douglas F. Easton, Charles Eaton, Paul Elliott, Evangelos Evangelou, Jessica D. Faul, Tatiana Foroud, Alison Goate, Jian Gong, Hans J. Grabe, Jeff Haessler, Christopher Haiman, Göran Hallmans, Anke R. Hammerschlag, Sarah E. Harris, Andrew Hattersley, Andrew Heath, Chris Hsu, William G. Iacono, Stavroula Kanoni, Manav Kapoor, Jaakko Kaprio, Sharon L. Kardia, Fredrik Karpe, Jukka Kontto, Jaspal S. Kooner, Charles Kooperberg, Kari Kuulasmaa, Markku Laakso, Dongbing Lai, Claudia Langenberg, Nhung Le, Guillaume Lettre, Anu Loukola, Jian’an Luan, Pamela A. F. Madden, Massimo Mangino, Riccardo E. Marioni, Eirini Marouli, Jonathan Marten, Nicholas G. Martin, Matt McGue, Kyriaki Michailidou, Evelin Mihailov, Alireza Moayyeri, Marie Moitry, Martina Müller-Nurasyid, Aliya Naheed, Matthias Nauck, Matthew J. Neville, Sune Fallgaard Nielsen, Kari North, Markus Perola, Paul D. P. Pharoah, Giorgio Pistis, Tinca J. Polderman, Danielle Posthuma, Neil Poulter, Beenish Qaiser, Asif Rasheed, Alex Reiner, Frida Renström, John Rice, Rebecca Rohde, Olov Rolandsson, Nilesh J. Samani, Maria Samuel, David Schlessinger, Steven H Scholte, Robert A. Scott, Peter Sever, Yaming Shao, Nick Shrine, Jennifer A. Smith, John M. Starr, Kathleen Stirrups, Danielle Stram, Heather M. Stringham, Ioanna Tachmazidou, Jean-Claude Tardif, Deborah J. Thompson, Hilary A. Tindle, Vinicius Tragante, Stella Trompet, Valerie Turcot, Jessica Tyrrell, Ilonca Vaartjes, Andries R van der Leij, Peter van der Meer, Tibor V. Varga, Niek Verweij, Henry Völzke, Nicholas J. Wareham, Helen R. Warren, David R. Weir, Stefan Weiss, Leah Wetherill, Hanieh Yaghootkar, Ersin Yavas, Yu Jiang, Fang Chen, Xiaowei Zhan, Weihua Zhang, Wei Zhao, Wei Zhao, Kaixin Zhou, Philippe Amouyel, Stefan Blankenberg, Mark J. Caulfield, Rajiv Chowdhury, Francesco Cucca, Ian J. Deary, Panos Deloukas, Emanuele Di Angelantonio, Marco Ferrario, Jean Ferrières, Paul W. Franks, Tim M. Frayling, Philippe Frossard, Ian P. Hall, Caroline Hayward, Jan-Håkan Jansson, J. Wouter Jukema, Frank Kee, Satu Männistö, Andres Metspalu, Patricia B. Munroe, Børge Grønne Nordestgaard, Colin N. A. Palmer, Veikko Salomaa, Naveed Sattar, Timothy Spector, David Peter Strachan, Understanding Society Scientific Group, EPIC-CVD, GSCAN, Consortium for Genetics of Smoking Behaviour, CHD Exome+ consortium, Pim van der Harst, Eleftheria Zeggini, Danish Saleheen, Adam S. Butterworth, Louise V. Wain, Goncalo R. Abecasis, John Danesh, Martin D. Tobin, Scott Vrieze, Dajiang J. Liu, Joanna M. M. Howson

**Affiliations:** 1 Department of Health Sciences, University of Leicester, Leicester, UK; 2 Department of Psychology, University of Minnesota, Minneapolis, MN USA; 3 Population Health and Immunity Division, The Walter and Eliza Hall Institute of Medical Research, 1G Royal Pde, 3052 Parkville, Australia; 4 Department of Medical Biology, University of Melbourne, Melbourne, 3010 Parkville Australia; 5 MRC/BHF Cardiovascular Epidemiology Unit, Department of Public Health and Primary Care, University of Cambridge, Cambridge, CB1 8RN, UK; 6 Institute for Behavioral Genetics, University of Colorado Boulder, Boulder, CO USA; 7 National Institute of Cardiovascular Diseases, Sher-e-Bangla Nagar, Dhaka, Bangladesh; 8 Department of Epidemiology and Biostatistics, Imperial College London, London, W2 1PG UK; 9 Department of Psychiatry, Washington University, St. Louis, MO USA; 10 Research Unit of Molecular Epidemiology, Helmholtz Zentrum München-German Research Center for Environmental Health, Neuherberg, Germany; 11 Centre for Cancer Genetic Epidemiology, Department of Public Health and Primary Care, University of Cambridge, Cambridge, CB1 8RN UK; 12 Department of Cardiology, Division Heart & Lungs, University Medical Center Utrecht, University of Utrecht, Utrecht, The Netherlands; 13 Durrer Center for Cardiovascular Research, Netherlands Heart Institute, Utrecht, The Netherlands; 14 Institute of Cardiovascular Science, Faculty of Population Health Sciences, University College London, London, UK; 15 Farr Institute of Health Informatics Research and Institute of Health Informatics, University College London, London, UK; 16 Department of Psychiatry, Washington University School of Medicine, St. Louis, MO USA; 17 Department of Neuroscience, Icahn School of Medicine at Mount Sinai, New York, NY USA; 18 Department of Biostatistics and Center for Statistical Genetics, University of Michigan, Ann Arbor, MI USA; 19 Julius Center for Health Sciences and Primary Care, University Medical Center Utrecht, 3508GA Utrecht, The Netherlands; 20 Center for Circulatory Health, University Medical Center Utrecht, 3508GA Utrecht, The Netherlands; 21 Department of Molecular, Cellular, and Developmental Biology, University of Colorado Boulder, Boulder, CO USA; 22 Lee Kong Chian School of Medicine, Nanyang Technological University, Singapore, 308232 Singapore; 23 Department of Cardiology, Ealing Hospital, Middlesex, UB1 3HW UK; 24 Imperial College Healthcare NHS Trust, London, W12 0HS UK; 25 Division of Cancer Epidemiology, German Cancer Research Centre (DKFZ), Heidelberg, Germany; 26 Cancer Epidemiology Group, University Medical Centre Hamburg-Eppendorf, University Cancer Centre Hamburg (UCCH), Hamburg, Germany; 27 Public Health Sciences Division, Fred Hutchinson Cancer Research Center, Seattle, WA USA; 28 Department of Epidemiology, University of Washington, Seattle, WA USA; 29 Centre for Cognitive Ageing and Cognitive Epidemiology, University of Edinburgh, Edinburgh, EH8 9JZ UK; 30 Psychology, University of Edinburgh, Edinburgh, EH8 9JZ UK; 31 Department of Medicine, Stanford University, Stanford, CA USA; 32 Department of Cardiology, University Medical Center Groningen, University of Groningen, Groningen, The Netherlands; 33 Department of Complex Trait Genetics, Center for Neurogenomics and Cognitive Research, Amsterdam Neuroscience, VU University Amsterdam, Amsterdam, Netherlands; 34 Institute of Cardiovascular and Medical Sciences, College of Medical, Veterinary and Life Sciences, University of Glasgow, Glasgow, UK; 35 Centre for Cancer Genetic Epidemiology, Department of Oncology, Cambridge Centre, University of Cambridge, Cambridge, CB1 8RN UK; 36 Department of Epidemiology and Biostatistics, Imperial College London, London, UK; 37 MRC-PHE Centre for Environment and Health, Imperial College London, London, W2 1PG UK; 38 National Institute for Health Research Imperial Biomedical Research Centre, Imperial College Healthcare NHS Trust and Imperial College London, London, UK; 39 UK Dementia Research Institute (UK DRI) at Imperial College London, London, UK; 40 Department of Hygiene and Epidemiology, University of Ioannina Medical School, Ioannina, Greece; 41 Department of Medical and Molecular Genetics, Indiana University School of Medicine, Indianapolis, IN USA; 42 Department of Neuroscience, Icahn School of Medicine at Mount Sinai, New York, NY USA; 43 Public Health Sciences Division, Fred Hutchinson Cancer Research Center, Seattle, WA USA; 44 Department of Psychiatry and Psychotherapy, University Medicine Greifswald, 17475 Greifswald, Germany; 45 Department of Preventive Medicine, Keck School of Medicine, University of Southern California, Los Angeles, CA United States; 46 Department of Public Health and Clinical Medicine, Nutritional research, Umeå University, Umeå, Sweden; 47 Centre for Genomic and Experimental Medicine, University of Edinburgh, Edinburgh, EH4 2XU UK; 48 Genetics of Complex Traits, University of Exeter Medical School, Exeter, UK; 49 University of Southern California, California, CA USA; 50 William Harvey Research Institute, Barts and The London School of Medicine and Dentistry, Queen Mary University of London, London, EC1M 6BQ UK; 51 Centre for Genomic Health, Queen Mary University of London, London, EC1M 6BQ UK; 52 Institute for Molecular Medicine Finland (FIMM), University of Helsinki, Helsinki, Finland; 53 Department of Public Health, University of Helsinki, Helsinki, Finland; 54 Department of Epidemiology, School of Public Health, University of Michigan, Ann Arbor, MI USA; 55 Oxford Centre for Diabetes, Endocrinology and Metabolism, University of Oxford, Oxford, UK; 56 Oxford National Institute for Health Research, Biomedical Research Centre, Churchill Hospital, Oxford, UK; 57 Department of Public Health Solutions, National Institute for Health and Welfare, FI-00271 Helsinki, Finland; 58 National Heart and Lung Institute, Imperial College London, London, W12 0NN UK; 59 Department of Biostatistics, University of Washington School of Medicine, Seattle, WA USA; 60 University of Eastern Finland, Finland, Finland; 61 MRC Epidemiology Unit, Institute of Metabolic Science, University of Cambridge School of Clinical Medicine, Cambridge, CB2 0QQ UK; 62 Department of Medical Microbiology, Immunology and Cell Biology, Southern Illinois University School of Medicine, Springfield, IL USA; 63 Montreal Heart Institute, Montreal, Quebec, H1T 1C8 Canada; 64 Department of Medicine, Faculty of Medicine, Universite de Montreal, Montreal, Quebec, H3T 1J4 Canada; 65 NIHR Biomedical Research Centre at Guy’s and St Thomas’ Foundation Trust, London, SE1 9RT UK; 66 MRC Human Genetics Unit, MRC Institute of Genetics and Molecular Medicine, University of Edinburgh, Edinburgh, UK; 67 Queensland Institute for Medical Research, Brisbane, Australia; 68 Department of Electron Microscopy/Molecular Pathology, The Cyprus Institute of Neurology and Genetics, 1683 Nicosia, Cyprus; 69 Estonian Genome Center, University of Tartu, Tartu, Estonia; 70 Institute of Health Informatics, University College London, London, UK; 71 Department of Epidemiology and Public health, University Hospital of Strasbourg, Strasbourg, France; 72 Institute of Genetic Epidemiology, Helmholtz Zentrum München - German Research Center for Environmental Health, Neuherberg, Germany; 73 Department of Medicine I, Ludwig-Maximilians-University Munich, Munich, Germany; 74 DZHK (German Centre for Cardiovascular Research), Partner Site Munich Heart Alliance, Munich, Germany; 75 Initiative for Noncommunicable Diseases, Health Systems and Population Studies Division, International Centre for Diarrhoeal Disease Research, Bangladesh (icddr,b) International Centre for Diarrhoeal Disease Research, Dhaka, Bangladesh; 76 Institute of Clinical Chemistry and Laboratory Medicine, University Medicine Greifswald, 17475 Greifswald, Germany; 77 DZHK (German Centre for Cardiovascular Research), Partner Site Greifswald, University Medicine, Greifswald, Germany; 78 Department of Clinical Biochemistry Herlev Hospital, Copenhagen University Hospital, Herlev Ringvej 74, DK-2730 Herlev, Denmark; 79 Department of Epidemiology, University of North Carolina, Chapel Hill, NC USA; 80 Survey Research Center, Institute for Social Research, University of Michigan, Ann Arbor, MI USA; 81 Istituto di Ricerca Genetica e Biomedica, Consiglio Nazionale delle Ricerche (CNR), Monserrato, Cagliari, Italy; 82 Department of Clinical Genetics, VU University Medical Centre Amsterdam, Amsterdam Neuroscience, Amsterdam, Netherlands; 83 International Centre for Circulatory Health, Imperial College London, London, UK; 84 Centre for Non-Communicable Diseases, Karachi, Pakistan; 85 Genetic and Molecular Epidemiology Unit, Lund University Diabetes Centre, Department of Clinical Sciences, Skåne University Hospital, Lund University, SE-214 28, Malmö, Sweden; 86 Department of Biobank Research, Umeå University, SE-901 87, Umeå, Sweden; 87 Departments of Psychiatry and Mathematics, Washington University St. Louis, St. Louis, MO USA; 88 University of North Carolina, Chapel Hill, NC USA; 89 Department of Public Health & Clinical Medicine, Section for Family Medicine, Umeå universitet, SE 90185 Umeå, Sweden; 90 Department of Cardiovascular Sciences, University of Leicester, Cardiovascular Research Centre, Glenfield Hospital, Leicester, LE3 9QP UK; 91 National Institute on Aging, National Institutes of Health, Bethesda, MD USA; 92 Department of Psychology, University of Amsterdam & Amsterdam Brain and Cognition, University of Amsterdam, Amsterdam, Netherlands; 93 Alzheimer Scotland Research Centre, University of Edinburgh, Edinburgh, EH8 9JZ UK; 94 Department of Haematology, University of Cambridge, Cambridge, CB2 0PT UK; 95 Department of Preventative Medicine, Keck School of Medicine, University of Southern California, Los Angeles, CA USA; 96 Wellcome Trust Sanger Institute, Hinxton, Cambridge, CB10 1SA UK; 97 Department of Medicine, Vanderbilt University, Nashville, TN USA; 98 Department of Cardiology, Division Heart and Lungs, University Medical Center Utrecht, Utrecht University, 3508GA Utrecht, The Netherlands; 99 Department of gerontology and geriatrics, Leiden University Medical Center, Leiden, The Netherlands; 100 Department of cardiology, Leiden University Medical Center, Leiden, The Netherlands; 101 Program in Medical and Population Genetics, Broad Institute of MIT and Harvard, 301 Binney Street, Cambridge, MA 02142 USA; 102 Institute for Community Medicine, University Medicine Greifswald, 17475 Greifswald, Germany; 103 Clinical Pharmacology, William Harvey Research Institute, Queen Mary University of London, London, EC1M 6BQ UK; 104 NIHR Barts Cardiovascular Biomedical Research Centre, Barts and The London School of Medicine and Dentistry, Queen Mary University of London, London, EC1M 6BQ UK; 105 Interfaculty Institute for Genetics and Functional Genomics, University Medicine and Ernst-Moritz-Arndt-University Greifswald, 17475 Greifswald, Germany; 106 Department of Neuroscience, Psychology and Behaviour, University of Leicester, Leicester, UK; 107 Department of Biomedical Engineering, The Pennsylvania State University, University Park, Pennsylvania, PA 16802 USA; 108 Institute of Personalized Medicine, Penn State College of Medicine, Hershey, PA USA; 109 Department of Clinical Science, Center for Genetics of Host Defense, University of Texas Southwestern, Dallas, TX USA; 110 Department of Cardiology, Ealing Hospital, London North West Healthcare NHS Trust, Middlesex, UB1 3HW UK; 111 Department of Biostatistics and Epidemiology, University of Pennsylvania, Pennsylvania, PA USA; 112 School of Medicine, University of Dundee, Dundee, UK; 113 Department of Epidemiology and Public Health, Institut Pasteur de Lille, Lille, France; 114 Department of General and Interventional Cardiology, University Heart Center Hamburg, Hamburg, Germany; 115 University Medical Center Hamburg Eppendorf, Hamburg, Germany; 116 William Harvey Research Institute, Barts and The London School of Medicine and Dentistry, Queen Mary University of London, London, EC1M 6BQ UK; 117 Princess Al-Jawhara Al-Brahim Centre of Excellence in Research of Hereditary Disorders (PACER-HD), King Abdulaziz University, Jeddah, 21589 Saudi Arabia; 118 National Institute for Health Research Blood and Transplant Research Unit in Donor Health and Genomics, Department of Public Health and Primary Care, University of Cambridge, Cambridge, CB1 8RN UK; 119 EPIMED Research Centre, Department of Medicine and Surgery, University of Insubria at Varese, Varese, Italy; 120 Department of Epidemiology, UMR 1027- INSERM, Toulouse University-CHU Toulouse, Toulouse, France; 121 Department of Nutrition, Harvard T. H. Chan School of Public Health, Boston, MA 02115 USA; 122 Division of Respiratory Medicine and NIHR Nottingham Biomedical Research Centre, University of Nottingham, Nottingham, UK; 123 Department of Public Health and Clinical Medicine, Skellefteå Research Unit, Umeå University, Umeå, Sweden; 124 Department of Cardiology, Leiden University Medical Center, Leiden, The Netherlands; 125 The Interuniversity Cardiology Institute of the Netherlands, Utrecht, The Netherlands; 126 UKCRC Centre of Excellence for Public Health, Queens, University, Belfast, Belfast, UK; 127 Medical Research Institute, University of Dundee, Ninewells Hospital and Medical School, Dundee, UK; 128 Institute of Cardiovascular and Medical Sciences, University of Glasgow, Glasgow, UK; 129 Department of Twin Research and Genetic Epidemiology, Kings College London, London, SE1 7EH UK; 130 Population Health Research Institute, St George!s, University of London, London, SW17 0RE UK; 131 Department of Genetics, University of Groningen, University Medical Center Groningen, Groningen, The Netherlands; 132 Department of Biostatistics and Epidemiology, Perelman School of Medicine, University of Pennsylvania, Pennsylvania, PA USA; 133 Center for Non-Communicable Diseases, Karachi, Pakistan; 134 National Institute for Health Research Leicester Respiratory Biomedical Research Centre, Glenfield Hospital, Leicester, UK

**Keywords:** Genetics, Addiction

## Abstract

Smoking is a major heritable and modifiable risk factor for many diseases, including cancer, common respiratory disorders and cardiovascular diseases. Fourteen genetic loci have previously been associated with smoking behaviour-related traits. We tested up to 235,116 single nucleotide variants (SNVs) on the exome-array for association with smoking initiation, cigarettes per day, pack-years, and smoking cessation in a fixed effects meta-analysis of up to 61 studies (up to 346,813 participants). In a subset of 112,811 participants, a further one million SNVs were also genotyped and tested for association with the four smoking behaviour traits. SNV-trait associations with *P* < 5 × 10^−8^ in either analysis were taken forward for replication in up to 275,596 independent participants from UK Biobank. Lastly, a meta-analysis of the discovery and replication studies was performed. Sixteen SNVs were associated with at least one of the smoking behaviour traits (*P* < 5 × 10^−8^) in the discovery samples. Ten novel SNVs, including rs12616219 near *TMEM182*, were followed-up and five of them (rs462779 in *REV3L*, rs12780116 in *CNNM2*, rs1190736 in *GPR101*, rs11539157 in *PJA1*, and rs12616219 near *TMEM182*) replicated at a Bonferroni significance threshold (*P* < 4.5 × 10^−3^) with consistent direction of effect. A further 35 SNVs were associated with smoking behaviour traits in the discovery plus replication meta-analysis (up to 622,409 participants) including a rare SNV, rs150493199, in *CCDC141* and two low-frequency SNVs in *CEP350* and *HDGFRP2*. Functional follow-up implied that decreased expression of *REV3L* may lower the probability of smoking initiation. The novel loci will facilitate understanding the genetic aetiology of smoking behaviour and may lead to the identification of potential drug targets for smoking prevention and/or cessation.

## Introduction

Smoking is a major risk factor for many diseases, including common respiratory disorders such as chronic obstructive pulmonary disease (COPD) [1, 2], cancer [3] and cardiovascular diseases [4], and is reported to cause 1 in 10 premature deaths worldwide [5]. A greater understanding of the genetic aetiology of smoking behaviour has the potential to lead to new therapeutic interventions to aid smoking prevention and cessation, and thereby reduce the global burden of such diseases.

Previous genome-wide association studies (GWASs) identified 14 common SNVs [1, 6–12] (with minor allele frequency, MAF >0.01) robustly associated with smoking behaviour-related traits (*P* < 5 × 10^−8^). The 15q25 (*CHRNA3/5-CHRNB4*) region has the largest effect, explaining ~1% and 4–5% of the phenotypic variance of smoking quantity [13] and cotinine, a biomarker of nicotine intake [14], respectively. Overall, genetic loci identified to date explain ~2% of the estimated genetic heritability of smoking behaviour [6], which is reported to be between 40–60% [15–17]. A recent study suggested that an important proportion (~3.3%) of the phenotypic variance of smoking behaviour-related traits was explained by rare nonsynonymous variants (MAF <0.01) [18]. Hence, well-powered studies of rare variants are needed.

To investigate the effect of rare coding variants on smoking behaviour, we studied 346,813 participants (of which 324,851 were of European ancestry) from 61 cohorts (Supp. Tables 1 and 2) at up to 235,116 SNVs from the exome array. As we had access to UK Biobank, we also interrogated SNVs present on the UK Biobank and UK BiLEVE Axiom arrays to identify additional associations across the genome beyond the exome array. To our knowledge, these datasets are an order of magnitude larger than the previous studies [6], and constitute the most powerful exome-array study of smoking behaviour to date.

## Materials and methods

### Participants

**Fig. 1:**
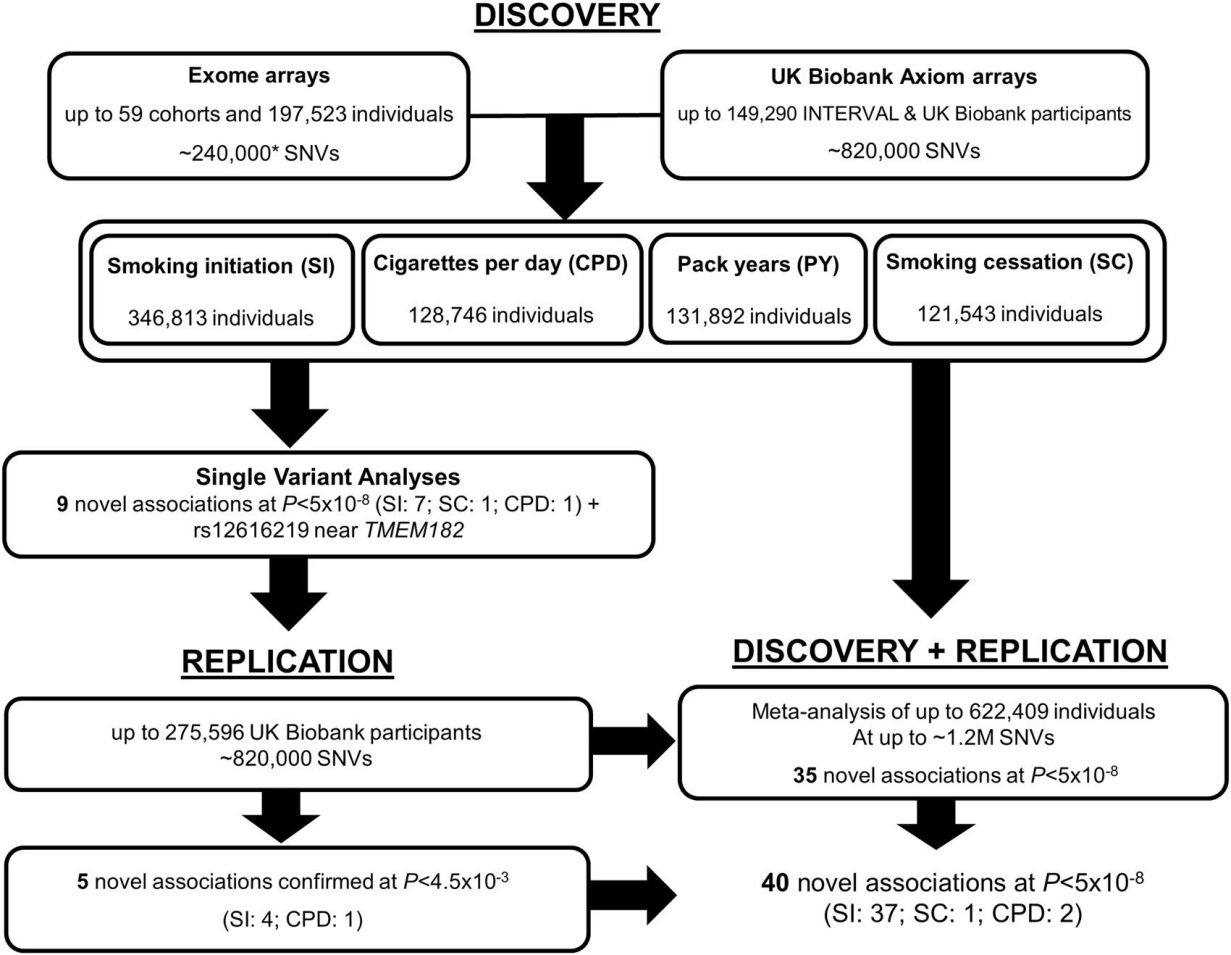
Study design including the discovery and replication stages. NB: Gene-based studies, conditional analyses, and replication in African American ancestry samples not shown here for clarity. *GFG and NAGOZALC studies contributed additional custom content

Our study combined study-level summary association data from up to 59 studies of European ancestry and two studies of South Asian ancestry from three consortia (Consortium for Genetics of Smoking Behaviour (CGSB), GWAS & Sequencing Consortium of Alcohol and Nicotine use (GSCAN) and the Coronary Heart Disease (CHD) Exome+ consortium), INTERVAL and UK Biobank. In total, up to 324,851 individuals of European ancestry and 21,962 South Asian individuals were analysed in the discovery stage (Fig. 1). Further information about the participating cohorts and consortia is given in Supp. Table 1 and the Supp. Material. All participants provided written informed consent and studies were approved by local Research Ethics Committees and/or Institutional Review boards.

### Phenotypes

We chose to analyse the following four smoking behaviour-related traits because of their broad availability in existing epidemiological and medical studies, as well as their biological relevance for addiction behaviours:

i. Smoking initiation (binary trait: ever vs never smokers). Ever smokers were defined as individuals who have smoked >99 cigarettes in their lifetime, which is consistent with the definition by the Centre for Disease Control [19];
ii. Cigarettes per day (CPD; quantitative trait: average number of cigarettes smoked per day by ever smokers);
iii. Pack-years (quantitative trait; Packs per day x Years smoked, with a pack defined as 20 cigarettes); years smoked is typically formed from age at smoking commencement to current age for current smokers or age at cessation for former smokers.
iv. Smoking cessation (binary trait: former vs current smokers).

In UK Biobank, phenotypes were defined using phenotype codes 1239, 1249, and 2644 for smoking initiation and smoking cessation, and 1239, 3436, 3456 for CPD and pack-years. CPD was inverse normal transformed in the CHD Exome+, INTERVAL and CGSB studies and categorised (1–10, 11–20, 21–30, and 31+ CPD) by the GSCAN studies and UK Biobank (Supp. Table 2). All studies performed an inverse normal transformation of pack-years. Summary statistics of study level phenotype distributions are provided in Supp. Table 1.

### Genotyping and quality control

Fifty-nine cohorts were genotyped using exome arrays (up to 235,116 SNVs) and two (UK Biobank and INTERVAL) were genotyped using Axiom Biobank Arrays (up to 820,000 SNVs; Supp. Table 2). In total, ~1.06M SNVs were analysed including ~64,000 SNVs on both the Axiom and Exome Arrays. Furthermore, two studies (NAGOZALC and GFG) genotyped their participants using arrays with custom content, increasing the total number of variants analysed to 1,207,583 SNVs. Individual studies performed quality control (QC; Supp. Material, Supp. Table 2) and additional QC was conducted centrally (i) to ensure alleles were consistently aligned, (ii) that there were no major sample overlaps between contributing studies, and (iii) variants conformed to Hardy–Weinberg equilibrium and call rate thresholds. We also examined the distribution of the effect sizes and test statistics across cohorts to ensure the test statistics were well-calibrated.

### Study level analyses

Each study (including the case-cohort studies [20]) undertook analyses of up to four smoking traits using RAREMETALWORKER [21] or RVTESTS [22] (Supp. Table 2), which generated single variant score statistics and their covariance matrices within sliding windows of 1Mb. CPD and pack-years were analysed using linear models or linear mixed models. Smoking initiation and smoking cessation were analysed using logistic models or linear mixed models. All studies adjusted each trait for age, sex, at least three genetic principal components and any study-specific covariates (Supp. Table 2). Chromosome X variants were analysed using the above-described approach, but coding males as 0/2. This coding scheme ensures that on average females and males have equal dosages and so is optimal for genes that are inactivated (due to X chromosome inactivation) and is valid for genes that do not undergo X chromosome activation. Males and females were analysed together adjusting for sex as a covariate.

### Single variant meta-analyses

Fixed effects meta-analyses across the individual contributing studies of single variant associations were undertaken using the Cochran-Mantel-Haenszel method in RAREMETAL. Z-score statistics were used in the meta-analysis to ensure that the association results are robust against potentially different units of measurement in the phenotype definitions across studies [23]. We performed genomic control correction on the meta-analysis results. Variants with *P* < 1 × 10^−6^ in tests of heterogeneity were excluded. Variants with *P* ≤ 5 × 10^−8^ were taken forward for replication. In addition, rs12616219 was also taken forward for replication as its *P*-value was very close to this threshold (smoking initiation, *P* = 5.49 × 10^−8^). None of the rare SNVs were genome-wide significant, therefore we also took forward the rare variant with the smallest association *P*-value, rs141611945 (*P* = 2.95 × 10^−7^; MAF <  0.0001).

### Replication and combined meta-analysis of discovery and replication data

As UK biobank genetic data were released in two phases, we took the opportunity to replicate findings from the discovery stage in a further 275,596 individuals made available in the phase two release of UK Biobank genetic data. To avoid potential relatedness between discovery and replication samples, the replication samples were screened and individuals with relatedness closer than second degree with the discovery sample in the UK Biobank were removed [24]. Phenotypes were defined in the same way as the discovery samples (described above). Since the exome array and the UK Biobank Axiom arrays do not fully overlap, we used both genotyped exome variants (approx. 64,000) as well as the additional ~90,000 well-imputed exome array variants from UK Biobank (imputation quality score >0.3) for replication of single variant and gene-based tests. The rare *ATF6* variant was absent from the UK Biobank array and is more prevalent in Africans (MAF = 0.01) than Europeans (MAF = 0.0007). Therefore, replication was sought in 1,437 individuals of African American-ancestry from the HRS and COGA studies. Analysis methods for replication cohorts were the same as for discovery cohorts, including methods to analyse chromosome X (Supp. Table 2). The criteria set for the replication were (i) the same direction of effect as the discovery analysis and (ii) *P* ≤ 0.0045 in the replication studies (Bonferroni-adjusted for eleven SNVs at α = 0.05).

Finally, in order to fully utilise all available data, we carried out a combined meta-analysis of the discovery and replication samples across the exome array content using the same protocols mentioned above.

### Conditional analyses

To identify conditionally independent variants within previously reported and novel loci a sequential forward stepwise selection was performed [25]. A 1 MB region was defined around the reported or novel sentinel variant (500 kb either side) and conditional analyses performed with all variants within the region. If a conditionally independent variant was identified, (*P* < 5 × 10^−6^; Bonferroni-adjusted for ~10,000 independent variants in the test region) the analysis was repeated conditioning on both the most significant conditionally independent variant and the sentinel variant. This stepwise approach was repeated (conditioning on the variants identified in current and earlier iterations) until there were no variants remaining in the region that were conditionally independent. The same protocol was followed for the novel SNVs identified in this study.

### Gene-based analyses

For discovery gene-based meta-analyses, we utilised three statistical methods as part of the RAREMETAL package: the Weighted Sum Test (WST) [26], the burden test [27] and the Sequence Kernel Association test (SKAT) [28]. EPACTS (v.3.3.0) [29] was used to annotate variants (for use in gene-based meta-analyses), as recommended by RAREMETAL. Two MAF cut-offs were used, one used low-frequency (MAF < 0.05) and rare variants, the second only used rare variants (MAF < 0.01). Nonsynonymous, stop gain, splice site, start gain, start loss, stop loss, and synonymous variants were selected for inclusion. A sensitivity analysis to exclusion of synonymous variants was also performed. Gene-level associations with *P* < 8 × 10^−7^ were deemed statistically significant (Bonferroni-adjusted for ~20,000 genes and three tests at α = 0.05). To examine if the gene associations were driven by a single variant, the gene tests were conducted conditional on the SNV with the smallest *P*-value in the gene, using the shared single variant association statistic and covariance matrices [21, 25].

### Mendelian randomization analyses

To evaluate the causal effect of SI and CPD on BMI, schizophrenia and educational attainment (EA), we conducted Mendelian randomization (MR) analyses using three complementary approaches available in MR-Base [30]: inverse variance weighted regression [31], MR-Egger [32, 33], and weigh**t**ed median [34]. We used both the previously reported smoking-associated SNVs and the SNVs from the current report (as provided in Tables 1–3 and Supp. Table 3) as instrumental variables. The BMI [35], schizophrenia [36] and educational attainment [37] data came from previously published publicly available data. To assess possible reverse causation, we also used outcome associated SNVs as instrumental variables and conducted MR analyses using SI and CPD as outcome. We considered *P* < 0.05/3 = 0.017 as statistically significant (Bonferroni-adjusted for three traits).

**Table 1:** Association results for SNVs identified in single variant association meta-analyses and taken forward to replication are provided

dbSNP ID (Exome ID)	Chr:Pos	EA/OA	Gene	Consequence	Trait	Discovery stage				Replication stage	
						N	EAF	DoE	*P*-value	Beta (SE)	*P*-value
rs141611945 (exm118559)	1:161771868	G/A	*ATF6*	Missense	CPD	128,746	0.0065% MAC = 9	+	2.95 × 10^−7^	0.184 (0.169)	*P = 0.276 in African American samples
rs1190736 ** (exm1659559)	X:136113464	A/C	*GPR101*	Missense	CPD (PY)	99,037 (96,824)	46.6% (47.0%)	-	**1.40** × **10**^**−11**^ (**4.98 x 10**^**−9**^)	−0.028 (0.0041) −0.027 (0.0049) −0.028 (0.0073)	All samples: **8.20 x 10**^**−12**^ (**2.70 x 10**^**−11**^) Males only: **1.90 x 10**^**−8**^ (**6.0 x 10**^**−8**^) Females only: **1.10 x 10**^**−4**^ (**7.1 x 10**^**−4**^)
rs462779 (exm572256)	6:111695887	A/G	*REV3L*	Missense	SI	346,682	80.1%	-	**4.52** × **10**^**−8**^	−0.023 (0.0034)	**9.7 x 10**^**−12**^
rs216195 (exm1276230)	17:2203167	G/T	*SMG6*	Missense	SI	335,406	27.3%	-	2.80 × 10^−8^	−0.008 (0.0029)	8.5 x 10^-3^
rs11539157 (exm1643833)	X:68381264	A/C	*PJA1*	Missense	SI	289,917	16.5%	+	**1.39** × **10**^−**11**^	0.022 (0.0026) 0.0158 (0.0033) 0.0185 (0.0039)	All samples: **5.40 x 10**^**−17**^ Males only: **1.30 x 10**^**−6**^ Females only: **2.20 x 10**^**−6**^
*Non-Exome-chip SNVs*											
rs12616219	2:104352495	A/C	*TMEM182*	Intergenic	SI	112,811	46.4%	-	**5.49** × **10**^**−8**^	−0.015 (0.0027)	**5.5 x 10**^**−8**^
rs1150691	6:28168033	G/A	*ZSCAN9*	Missense	SI	112,811	34.8%	-	4.95 × 10^−8^	−0.007 (0.0028)	8.0 x 10^-3^
rs2841334	9:128122320	A/G	*GAPVD1*	Intronic	SI	112,811	20.9%	-	2.28 × 10^−8^	−0.009 (0.0033)	7.5 x 10^-3^
rs202664	22:41813886	C/T	*TOB2*	Intergenic	SC	51,043	19.9%	-	1.02 × 10^−**8**^	−0.011 (0.0050)	2.1 x 10^-2^
rs11895381	2:60053727	A/G	*BCL11A*	Intergenic	SI	112,811	34.2%	-	5.61 × 10^−9^	−0.007 (0.0028)	1.2 x 10^-2^
rs12780116	10:104821946	A/G	*CNNM2*	Intronic	SI	112,811	13.9%	+	**9.19** × **10**^−**10**^	0.017 (0.0039)	**1.1 x 10**^**−5**^

Novel smoking trait associated SNVs that replicated with *P* < 0.005 and had consistent direction of effect in discovery and replication are highlighted in bold. The replication sample size for smoking initiation (SI), cigarettes per day (CPD), pack-years (PY), and smoking cessation (SC) were 275,596, 80,015, 78,897, and 123,851 respectively. Chromosome (Chr) and position (Pos) for hg19 build 37. *EA* effect allele, *OA* other allele, *Gene* closest gene, *N* number of individuals, *EAF* effect allele frequency in the pooled samples, *MAC* minor allele count, *DoE* direction of effect, *SE* standard error. All SNVs had heterogeneity *P* > 0.02 in the discovery stage. *Replication was sought in 1,437 individuals of African American-ancestry from the HRS and COGA studies; **The beta(se) for the association of rs1190736 with PY in the replication stage was −0.026 (0.0039)

**Table 2:** Association results for novel SNVs identified in the combined meta-analysis of the discovery and replication cohorts

dbSNP ID (Exome-chip ID)	Chr:Pos	EA/OA	Gene	Consequence	Trait	EAF	Beta (se) in replication stage	*P*-value in combined meta-analysis (*P*-value in Discovery/Replication stage)	Notes
*Combining only genotyped Exome-chip content on the Axiom array*									
rs1514175	1:74991644	G/A	*TNNI3K*	Intronic	SI	0.57	−0.011 (0.003)	**5.42** × **10**^−**9**^ (9.03 x 10^−5^/1.0 x 10^−5^)	Previously associated with BMI
rs7096169	10:104618695	G/A	*BORCS7* (*CNNM2*^*#*^ in Table 1)	Intronic	SI	0.31	0.016 (0.003)	**2.17** × **10**^−**13**^ (3.38 × 10^−7^/7.3 × 10^−9^)	*r*^2^ = 0.28 between rs7096169 and rs12780116 (Table 1) in 1000 Genomes EUR. Previously associated with Schizophrenia. rs7096169 an eQTL for *ARL3*, BORCS7, and *AS3MT* in ≥1 of the brain tissues in GTEx
rs2292239	12:56482180	G/T	*ERBB3*	Intronic	SI	0.66	0.0121 (0.003)	**2.78** × **10**^−**8**^ (7.56 × 10^−5^/1.5 × 10^−5^)	Previously associated with type-1 diabetes and years of educational attainment. rs2292239 is an eQTL for *RPS26* and *SUOX* in ≥4 of the brain tissues in GTEx
rs216195	17:2203167	G/T	*SMG6*^*#*^	Missense	SI	0.29	−0.0076 (0.003)	**2.41** × **10**^−**9**^ (2.80 × 10^−8^/8.5 × 10^−3^)	Same SNV as in Table 1
*Combining well-imputed Exome-chip content on the Axiom array*									
rs2960306 (exm383568)	4:2990499	T/G	*GRK4*	Missense	CPD	0.34	−0.024 (0.005)	**1.06** × **10**^−**9**^ (3.99 × 10^−5^/3.8 × 10^−6^)	rs2960306 is an eQTL for *GRK4* in four of the brain tissues in GTEx
rs4908760	1:8526142	A/G	*RERE*	Intronic	SI	0.35	0.0078 (0.003)	**1.76** × **10**^−**8**^ (3.36 × 10^−6^/4.7 × 10^−3^)	Previously associated with Vitiligo
rs6692219 (exm127721)	1:179989584	C/G	*CEP350*	Missense	SI	0.028	−0.0257 (0.008)	**4.69** × **10**^−**9**^ (1.08 × 10^−6^/1.3 × 10^−3^)	
rs11971186	7:126437897	G/A	*GRM8*	Intronic	SI	0.20	−0.0080 (0.003)	**1.45** × **10**^−**8**^ (1.38 × 10^−6^/3.9 × 10^−3^)	
rs150493199 (exm249655)	2:179721072	A/T	*CCDC141*	Missense	SC	0.0098	0.048 (0.134)	**1.28** × **10**^−**8**^ (6.45 × 10^−8^/0.72)	
*Non-Exome-chip SNVs*									
rs3001723	1:44037685	A/G	*PTPRF*	Intronic	SI	0.21	0.0159 (0.003)	**6.64** × **10**^−**11**^ (0.00015/4.1 × 10^−8^)	Previously associated with Schizophrenia and Years of educational attainment
rs1937455	1:66416939	G/A	*PDE4B*	Intronic	SI	0.30	−0.0146 (0.0027)	**1.23** × **10**^−**9**^ (0.00073/5.6 × 10^−8^)	
rs72720396	1:91191582	G/A	*BARHL2*	Intergenic	SI	0.16	−0.0150 (0.003)	**9.86** x **10**^−**9**^ (5.63 × 10^−5^/1.9 × 10^−6^)	
rs6673752	1:154219177	C/G	*UBAP2L*	Intronic	SI	0.055	−0.027 (0.004)	**1.1** × **10**^−**11**^ (NA/1.1 × 10^−11^)	
rs2947411	2:614168	G/A	*TMEM18*	Intergenic	SI	0.83	0.0189 (0.004)	**4.97** × **10**^−**10**^ (0.00017/7.1 × 10^−8^)	Previously associated with BMI
rs528301	2:45154908	A/G	*SIX3*	Intergenic	SI	0.38	0.0136 (0.002)	**4.12** × **10**^−**11**^ (1.77 × 10^−6^/3.8 × 10^−7^)	
rs6738833	2:104150891	T/C	*TMEM182*^*#*^	Intergenic	SI	0.33	−0.018 (0.003)	**8.66** × **10**^−**14**^ (1.63 × 10^−6^/4.4 × 10^−11^)	*r*^2^ = 0.69 between rs6738833 and rs12616219 (Table 1) in European samples of the 1000 Genomes Project
rs13026471	2:137564022	T/C	*THSD7B*	Intronic	SI	0.18	0.0127 (0.003)	**2.45** × **10**^−**8**^ (0.00028/3.0 x 10^−5^)	
rs6724928	2:156005991	C/T	*KCNJ3*	Intergenic	SI	0.32	−0.011 (0.003)	**4.47** × **10**^−**8**^ (0.0019/4.8 × 10^−5^)	
rs13022438	2:162800372	G/A	*SLC4A10*	Intronic	SI	0.27	0.0146 (0.003)	**1.41** × **10**^−**11**^ (0.0005/8.1 × 10^−8^)	
rs1869244	3:5724531	A/G	*LOC105376939*	Intergenic	SI	0.32	0.0123 (0.003)	**2.76** × **10**^−**9**^ (0.00040/4.1 x 10^−6^)	
rs35438712	3:85588205	T/C	*CADM2*	Intronic	SI	0.25	0.017 (0.003)	**1.99** × **10**^−**13**^ (1.15 × 10^−5^/3.2 × 10^−10^)	
rs6883351	5:22193967	T/C	*CDH12*	Intronic	SI	0.34	0.0129 (0.003)	**4.69** × **10**^**−8**^ (0.0010/1.4 × 10^−6^)	
rs6414946	5:87729711	C/A	*TMEM161B*	Intronic	SI	0.32	−0.0137 (0.003)	**5.27** × **10**^−**10**^ (3.63 × 10^−5^/2.8 × 10^−7^)	
rs11747772	5:166992708	C/T	*TENM2*	Intronic	SI	0.25	0.0144 (0.003)	**6.20** × **10**^−**9**^ (0.011/2.2 × 10^−7^)	
rs9320995	6:98726381	G/A	*POU3F2*	Intergenic	SI	0.18	0.0150 (0.003)	**1.70** × **10**^−**8**^ (0.00079/6.1 × 10^−7^)	
rs10255516	7:1675621	G/A	*ELFN1*	Intergenic	SI	0.33	−0.0139 (0.003)	**2.86** × **10**^−**10**^ (0.0021/1.8 × 10^−7^)	
rs10807839	7:3344629	G/A	*SDK1*	Intronic	SI	0.19	0.0162 (0.003)	**8.93** × **10**^−**11**^ (0.0026/4.4 × 10^−8^)	
rs6965740	7:117514840	T/G	*CTTNBP2*	Intergenic	SI	0.31	−0.0126 (0.003)	**9.66** × **10**^−**9**^ (5.56 × 10^−6^/2.8 × 10^−6^)	
rs11776293	8:27418429	T/C	*EPHX2*	Intronic	SI	0.12	−0.0200 (0.003)	**2.23** × **10**^−**12**^ (0.00011/8.9 × 10^−9^)	rs11776293 is an eQTL for *CHRNA2* in cerebellum in GTEx
rs1562612	8:59817068	G/A	*TOX*	Intronic	SI	0.35	−0.0112 (0.003)	**1.15** × **10**^−**9**^ (1.42 × 10^−5^/2.9 × 10^−5^)	
rs3857914	8:93184065	C/T	*RUNX1T1*	Intergenic	SI	0.19	0.0157 (0.003)	**1.54** × **10**^−**9**^ (0.065/7.1 × 10^−8^)	
rs2799849	9:86752641	C/T	*RMI1*	Intergenic	SI	0.22	−0.0156 (0.003)	**1.94** × **10**^−**8**^ (0.026/4.8 × 10^−8^)	
rs6482190	10:22037809	A/G	*LOC107984214*	Intronic	SI	0.17	0.0146 (0.003)	**8.85** × **10**^−**9**^ (0.0021/9.5 × 10^−7^)	
rs4523689	11:7950797	G/A	*OR10A6*	Intergenic	SI	0.27	−0.012 (0.003)	**7.77** × **10**^−**9**^ (0.00030/2.2 × 10^−5^)	
rs933006	13:38350193	A/G	*TRPC4*	Intronic	SI	0.32	−0.0143 (0.003)	**3.50** × **10**^−**8**^ (0.022/9.6 × 10^−8^)	
rs557899	15:47643795	A/C	*SEMA6D*	Intronic	SI	0.26	0.0157 (0.003)	**2.99** × **10**^−**13**^ (4.46 × 10^−5^/1.0 × 10^−8^)	
rs76608582	19:4474725	A/C	*HDGFRP2*	Intronic	SI	0.029	−0.0360 (0.007)	**8.50** × **10**^−**9**^ (0.012/4.3 × 10^−8^)	

Chromosome (Chr) and position (Pos) for each SNV is given for hg19 build 37. Only SNVs reaching genome-wide significance (*P* < 5 × 10^-8^, in bold) in the combined meta-analysis are shown. Magnitude of the effect size estimates are not presented as traits were transformed in differently by the three consortia analysed. SNVs identified in the discovery stage of this study (see Table 1) are denoted #. The discovery sample size for smoking initiation (SI), CPD, pack-years (PY), and smoking cessation (SC) were 346,813, 128,746, 131,892, and 121,543, respectively; and the replication sample size for SI, CPD, PY, and SC were 275,596, 80,015, 78,897, and 123,851, respectively. NB: rs6673752 (intronic to *UBAP2L*) was not available in the discovery cohorts. *EA* effect allele, *OA* other allele, *Beta(se)* beta and standard error for association in the replication stage. All SNVs had heterogeneity *P* > 0.0001

Bold font highlights the genome-wide significant *P*-values from the meta-analysis of discovery plus replication studies

**Table 3:** Results from conditional analyses at previously reported smoking behaviour loci

Gene region	dbSNP ID	Chr:Pos	EA/OA	Consequence	Trait	EAF	*P* (unconditional)	SNV(s) conditioned on	Discovery Conditional *P* [DoE]	Conditional *P* in replication [DoE]
19q13 (*RAB4B*)	rs8102683	19:41363765	C/T	Intergenic	CPD	74.8%	**4.53** × **10**^−**16**^	rs7937	**1.44** × **10**^−**13**^ [+]	3.5 **×** 10^-4^ [+]
	rs28399442	19:41354458	A/C	Intronic (*CYP2A6*)	CPD	1.3%	**2.27** × **10**^−**12**^	rs7937, rs8102683	**2.63** × **10**^−**12**^ [+]	**8.1** × **10**^−**14**^ [+]
	rs3865453	19:41338556	T/C	Intergenic	CPD	6.54%	**2.96** × **10**^−**12**^	rs7937, rs8102683, rs28399442	**4.96** × **10**^−**10**^ [−]	**2.3** × **10**^−**13**^ [−]
*TEX41-PABPC1P2*	rs11694518	2:146125523	T/C	Intergenic	SI	29.5%	**2.90** × **10**^−**9**^	rs10193706	3.43 × 10^−7^ [−]	**4.0** × **10**^−**31**^ [−]
15q25 (*CHRNA3*)	rs938682	15:78882925	A/G	Intronic (*CHRNA3*)	CPD	76.4%	**1.83** × **10**^−**69**^	rs1051730	**7.77** × **10**^−**21**^ [+]	**1.0** × **10**^−**13**^ [+]

SNVs with *P* < 5 × 10^−8^ are highlighted in bold. The discovery sample size for smoking initiation (SI) and CPD was 346,813 and 128,746, respectively. The replication sample size for SI and CPD were 275,596 and 80,015, respectively. *Chr* Chromosome, *Pos* position for hg19 build 37, *EA* effect allele, *OA* other allele, *EAF* effect allele frequency in the pooled samples, *DoE* Direction of effect

### In silico functional follow up of associated SNVs

To identify whether the (replicated) SNVs identified here affected other traits, we queried the GWAS Catalog [38] (version: e91/28/02/2018, downloaded on 01/03/18) for genome-wide significant (*P* < 5 × 10^−8^) associations using all proxy SNVs (*r*^2^ ≥ 0.8) within 2 Mb of the top variant in our study.

eQTL lookups were carried out in the 13 brain tissues available in GTEx V7 [39], Brain xQTL (dorsolateral prefrontal cortex) [40] and BRAINEAC [41] databases, all of which had undergone QC by the individual studies. We did not perform additional QC on these data. In brief, GTEx used Storey’s q-value method to correct the FDR for testing multiple transcripts based upon the empirical *P*-values for the most significant SNV for each transcript [43, 42]. BRAINEAC calculated the number of tests per transcript and used Benjamini–Hochberg procedure to calculate FDR per transcript using a FDR < 1% as significant. BRAINxQTL used *P* < 8 × 10^−8^ as a cut-off for significance for any given transcript. SNVs that met the study specific significance and FDR thresholds, which were in LD (*r*^2^ > 0.8 in 1000 Genomes Europeans) with the top eQTL or the sentinel eQTL for a given tissue/transcript combination were considered significant. The genes implicated by these eQTL databases and/or coding changes (e.g., missense and nonsense SNVs) were put into ConsensusPathDB [44] to identify whether these genes were over-represented in any known biological pathways. Replicated missense SNVs were also put into PolyPhen-2 [45] and FATHMM (unweighted) [46] to obtain variant effect prediction.

## Results

### Single variant associations

**Fig. 2:**
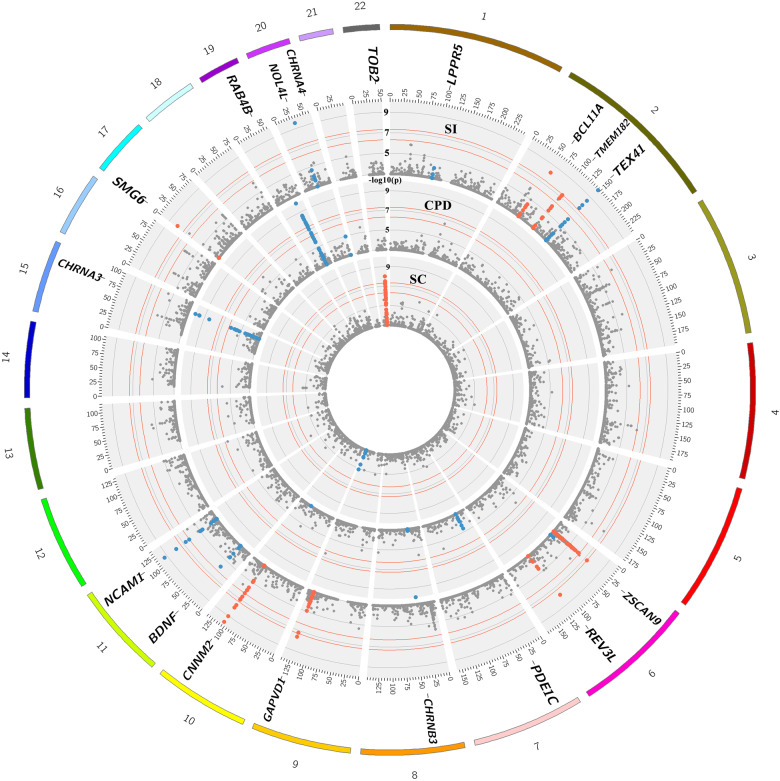
A concentric Circos plot of the association results for smoking initiation (SI; outer ring), cigarettes per day (CPD) and smoking cessation (SC; inner ring) for chromosomes 1–22 (Pack-years results, which can be found in Supp. Figure 1, are omitted for clarity). Each dot represents a SNV, with the X and Y axes corresponding to genomic location in Mb and -log_10_*P*-values, respectively. Labels show the nearest gene to the novel sentinel variants identified in the discovery stage and taken forward to replication. The top signals were truncated at 10^−10^ for clarity. Novel and previously reported signals are highlighted in red and dark blue, respectively. Grey rings on the y-axis increase by increments of 2 (initial ring corresponding to *P* = 0.001, then 0.00001 etc.); and the outer and inner red rings correspond to the genome-wide significance level (*P* = 5 × 10^−8^) and *P* = 5 × 10^−7^, respectively. Image was created using Circos (v0.65)

In the discovery meta-analyses, we identified 15 common SNVs that were genome-wide significant (*P* < 5 × 10^-8^) for one or more of the smoking behaviour traits, of which 9 were novel (Table 1, Supp. Table 3). Seven novel loci were identified for smoking initiation, one for both CPD and pack-years and one for smoking cessation (Figs. 1, 2, Table 1 and Supp. Figure 1). Results for the significant loci were consistent across participating cohorts and there was at least nominal evidence of association (*P* < 0.05) at the novel loci within each of the contributing consortia (Supp. Table 4). Full association results for all novel SNVs across the four traits are provided in Supp. Table 5. No rare variants were genome-wide significant; the rare variant with the smallest *P*-value was a missense variant in *ATF6*, rs141611945 (MAF < 0.0001, CPD *P* = 2.95 × 10^−7^).

Eleven SNVs (including rs12616219 near *TMEM182* with *P* = 5.49 × 10^−8^, and the rare variant, rs141611945) were taken forward for replication in independent samples (Table 1). The latest release of European UK Biobank individuals not included in the discovery stage (smoking initiation, *n* = 275,596; smoking cessation *n* = 123,851; CPD *n* = 80,015; pack-years *n* = 78,897), was used for replication of the common variants (Fig. 1). Five of the common variants replicated (four for smoking initiation and one with CPD and pack-years) at *P* < 0.0045. Two coding variants (rs11539157, rs1190736) were predicted to be ‘probably damaging’ by PolyPhen-2 and FATHMM. The remaining five SNVs were at least nominally associated (*P* < 0.01) in the replication samples and had consistent direction of effect across discovery and replication. Replication for the rare variant rs141611945 could not be carried out in UK Biobank as the SNV nor its proxies (*r*^2^ > 0.3) were available. Thus we initiated replication in African American samples of the COGA (*n* = 476) and HRS (*n* = 961) cohorts (overall MAF≈0.01). The direction of effect was consistent in the two replication cohorts and consistent with the discovery meta-analysis but a meta-analysis of the two replication cohorts yielded a *P* = 0.28. Further data are required to replicate this association.

We also performed a meta-analysis combining the discovery and replication samples (up to 622,409 individuals). LD score regression showed that the λ (intercept) for all traits was ~1.00, which indicated that confounding factors inflating the results was not an issue [47, 48]. The combined analysis identified 35 additional novel SNV-smoking trait associations, 33 with smoking initiation, one with CPD and one with smoking cessation at *P* < 5 ×10^-8^ (Table 2). We note that among our four SNVs that did not replicate, rs216195 (in *SMG6*) was genome-wide significant in the combined meta-analysis of discovery and replication studies (*P* = 2.41 × 10^−9^; Table 2).

We also calculated the phenotypic variance explained for novel and known variants. Results can be found in the ‘Calculation of Phenotypic Variance Explained’ section in the Supplementary Material.

### Associations at known smoking behaviour loci

We assessed evidence for associations at the 14 SNVs previously reported for smoking behaviour-related traits. Seven were genotyped on the exome array and proxies (*r*^2^ > 0.3; ±2 Mb) were identified for the remaining seven (Supp. Table 3). All showed nominal evidence of association at *P* < 0.05 and six of these were genome-wide significant in the meta-analysis of the trait for which it was previously reported (Supp. Tables 3 and 5).

Conditional analyses identified five independent associations within three previously reported loci and all five replicated (Table 3). At the 19q13 (*RAB4B*) locus, there were three variants in or near *CYP2A6* associated with CPD independently of the established variant (rs7937) and each other: rs8102683 (conditional *P* = 4.53 × 10^−16^), rs28399442 (conditional *P* = 2.63 × 10^−12^) and rs3865453 (conditional *P* = 4.96 × 10^−10^) and rs28399442 was a low-frequency variant. The same SNVs also showed evidence of independent effects with pack-years, albeit with larger *P*-values (*P* < 5 × 10^−6^; Supp. Table 5). At the *TEX41/PABPC1P2* locus, rs11694518 (conditional *P* = 3.43 × 10^−7^) was associated with smoking initiation independently of the established variant (rs10427255). At 15q25, rs938682 (*P* = 7.78 × 10^−21^) was associated with CPD independently of the established variant (rs1051730) and (in agreement with a previous report [49]) is an eQTL for *CHRNA5* in brain putamen basal ganglia tissues in GTEx.

### Gene-based association studies

Gene-based collapsing tests using MAF < 0.01 variants, did not identify any associated genes at the pre-specified *P* < 8 × 10^−7^ threshold. Of the top four gene associations, three were novel (*CHRNA2*, *MMP17*, and *CRCP*) and one was known (*CHRNA5*), and had *P* < 7 × 10^−4^, with CPD and/or pack-years (Supp. Table 6**)**. Analyses conditional on the variant with the smallest *P*-value in the gene, revealed the associations at *CHRNA2*, *MMP17* and *CRCP* were due to more than one rare variant (conditional *P* < 0.05; Supp. Table 6). In contrast, the *CHRNA5* gene association was attributable to a single variant (rs2229961).

### Mendelian randomization analyses

We conducted MR analyses to elucidate the potential causal impact of SI and CPD on BMI, schizophrenia and EA using the MR-Egger, median weighted and inverse variance weighted methods. We found a causal association between SI and EA using both the median weighted and inverse variance weighted methods (*P* < 0.0001; Supp. Table 7) but not with MR-Egger (*P* = 0.2). There was an association of SI with BMI using MR-Egger only (*P* = 0.01; Supp. Table 7), but there was evidence of horizontal pleiotropy (*P* = 0.001) and no support from the other methods. Similarly, increased CPD was only associated with reduced BMI using the weighted median approach (*P* = 0.009) and not the other methods (*P* > 0.017). We also tested if schizophrenia, EA or BMI causally influence CPD or SI using SNVs associated with schizophrenia, EA and BMI, respectively, as instrumental variables. No evidence of such reverse causation was found (Supp. Table 7). These results were consistent with previous analyses [50]. There was no evidence of a causal effect of SI on schizophrenia, or CPD on educational attainment (Supp. Table 7).

### Functional characterization of novel loci

Using proxies with r^2^≥0.8 in 1000 Genomes Europeans, we queried the GWAS catalogue [38] (*P* ≤ 5 × 10^−8^) for pleiotropic effects of our novel sentinel SNVs. Two, rs11539157 and rs3001723 were previously associated with schizophrenia [36], suggesting shared biological pathways between schizophrenia and smoking behaviours (Table 2). This fits with the known association of smoking with schizophrenia [51]. Two, rs1514175 and rs2947411 have previously been associated with BMI [52], and extreme obesity [53].

eQTL lookups in GTEx V7 (13 Brain tissues with ≥80 samples) [39], Brain xQTL [40] and BRAINEAC [41] databases revealed that the A allele at rs462779, which decreases risk of smoking initiation, also decreased expression of *REV3L* in cerebellum in GTEx (A allele *P* = 4.8x10^-8^; β = −0.40) and was in strong LD with the top eQTL for *REV3L* in cerebellum (*r*^2^ = 0.86 with rs9487668 in 1000 Genomes Europeans). The smoking initiation-associated SNV, rs12780116, was an eQTL for *BORCS7* in four brain tissues, and *NT5C2* in the cerebellar hemisphere (A allele *P* = 4.5 × 10^−7^; β = −0.32) and the cerebellum (*P* = 5.6 × 10^−6^; β = −0.415; in strong LD with the top eQTL, *r*^2^ = 0.97 with rs11191546). The G allele of a second variant in the region, rs7096169 (intronic to *BORCS7* and only in weak LD with rs12780116, *r*^2^ = 0.18 in 1000G Europeans) increases smoking initiation and reduces expression of *BORCS7* and *AS3MT* in eight brain tissues (including dorsolateral prefrontal cortex in the Brain xQTL and was the top *BORCS7* eSNP in GTEx in the Cerebellar Hemisphere, Cerebellum, and Spinal cord cervical-C1). The same variant also reduced expression of *ARL3* in cerebellum in GTEx (Table 2).

Biological pathway enrichment analyses carried out in ConsensusPathDB [44] using the genes implicated by the eQTL databases (Table 2) and/or a coding SNVs (i.e., *PJA1*, *GPR101*) showed that the (i) pyrimidine metabolism and (ii) activation of nicotinic acetylcholine receptors pathways are enriched for these smoking behaviour associated genes (false discovery rate <0.01; *P* < 0.0001).

## Discussion

Smoking is the most important preventable lifestyle risk factor for many diseases, including cancers [3, 54], heart disease [4, 55] and many respiratory diseases such as COPD [1, 2]. Not initiating is the best way to prevent smoking-related diseases and genetics can play a considerable part in smoking behaviours including initiation. We have performed the largest exome-wide genetic association study of smoking behaviour-related traits to date involving up to 622,409 individuals, and identified and replicated five associations, including two on the X-chromosome (Table 1). We identified a further 35 novel associations in a meta-analysis of discovery and replication cohorts (Table 2). We validated 14 previously reported SNV-smoking trait associations (Supp. Table 3) and identified secondary independent associations at three loci, including three in the 19q13 region (rs8102683, rs28399442, and rs3865453; Table 3).

Gene-based tests improve power by aggregating effects of rare variants. While no genes reached our Bonferroni-adjusted *P*-value threshold, we identified three candidate genes with multiple rare variant associations for future replication: calcitonin gene-related peptide-receptor component (*CRCP*) with CPD and *CHRNA2* and *MMP17* with pack-years (Supp. Table 6; also see ‘Genes of Interest’ section in Supp. Material). *CRCP*’s protein product is expressed in brain tissues amongst others and functions as part of a receptor complex for a neuropeptide that increases intracellular cyclic adenosine monophosphate levels [56]. *MMP17* encodes a matrix metalloproteinase that is also expressed in the brain and is a member of the peptidase M10 family, and proteins in this family are involved in the breakdown of extracellular matrix in normal physiological processes [57]. Given, we were not able conclusively to identify rare variant associations, even larger studies, are required to identify rare variants associated with smoking behaviours. In addition, phenotypes such as cotinine levels [58] and nicotine metabolism speed [59] could be interrogated using methods such as MTAG [60] to improve power.

As recommended by UK Biobank, we analysed UK Biobank samples by adjusting for genotyping array because a subset of (extreme smokers in) UK Biobank were genotyped on a different array (UK BiLEVE). However, this adjustment could potentially introduce collider bias in analyses of smoking traits. Given that the UK BiLEVE study is relatively small compared to the full study, and the genetic effect sizes for smoking-associated variants are small, we expect the influence of collider bias to be small [61]. Nevertheless, we performed sensitivity analyses to assess the impact of collider bias. Firstly, we performed a meta-analysis excluding the UK BiLEVE samples, and secondly, we re-analysed UK Biobank without adjusting for genotype array. As expected, the estimated genetic effects from these additional analyses were very similar to our reported results suggesting collider bias is not a concern (Suppl. Table 8).

Follow-up of the replicated SNVs in the literature and eQTL databases implicated some potentially interesting genes: *NT5C2* is known to hydrolyse purine nucleotides and be involved in maintaining cellular nucleotide balance, and was previously associated with schizophrenia [62]. *REV3L*, encodes the catalytic subunit of DNA polymerase ζ (zeta) which is involved in translesion DNA synthesis. Previously, polymorphisms in a microRNA target site of *REV3L* were shown to be associated with lung cancer susceptibility [63]. We showed that decreased expression of *REV3L* may also lower the probability of smoking initiation. The SNV, rs11776293, intronic in *EPHX2*, was associated with reduced SI in the combined meta-analysis, and is in LD with rs56372821 (*r*^2^ = 0.83), which is associated with reduced cannabis use disorder [64]. rs216195 (in *SMG6*) was genome-wide significant in the discovery and the combined meta-analysis. *SMG6* is a plausible candidate gene as it was previously shown to be less methylated in current smokers compared to never smokers [65]. The combined meta-analysis also identified a rare missense variant in *CCDC141*, rs150493199 (MAF < 0.01; Table 2). Coding variants in *CCDC141* were previously associated with heart rate [66] and blood pressure [67, 68].

Smoking behaviours represent a complex phenotype that are linked to an array of socio-cultural and familial, as well as genetic determinants. Kong et al., recently reported that ‘genetic-nurture’ i.e., effects of non-transmitted parental alleles, affect educational attainment [69]. They also show that there is an effect of educational attainment and genetic nurture on smoking behaviour. Four of our sentinel SNVs (or a strong proxy; *r*^2^ > 0.8) were associated with years of educational attainment [37] (rs2292239, rs3001723 (*P* < 5 × 10^−8^), rs9320995 (*P* = 8.90 x 10^−7^), and rs13022438 (*P* = 3.79 × 10^−6^), in agreement with this paradigm and our MR analyses indicated that initiating smoking reduced years in education. Future family studies will be required to disentangle how much of the variance explained in the current analysis is due to direct versus genetic nurturing effects.

Our study primarily focused on European ancestry, but we also included two non-European studies but these non-European studies lacked statistical power on their own to identify ancestry-specific effects. Therefore, we did not perform ancestry-specific meta-analyses. Nevertheless, our results offered cross ancestry replication. One of the associations identified in the conditional analyses, rs8102683 (near *CYP2A6*), confirmed an association with CPD that was previously identified by Kumasaka et al. in a Japanese population [70] but this is the first time it was associated in Europeans (rs8102683 is also correlated with rs56113850 (*r*^2^ = 0.43), a SNV identified previously by Loukola et al. [59] in a genetic association study of nicotine metabolite ratio in Europeans). As more non-European studies become available, it would be of great interest to perform non-European ancestry studies, in order to fine-map causal variants for smoking-related traits.

CPD and pack-years are two correlated measures of smoking. In the ~40,000 individuals from UK Biobank with CPD and pack-years calculated, correlation between CPD and pack-years was 0.640. Interestingly, while pack-years was inversely correlated with smoking cessation (−0.18) i.e., the more years a smoker has been smoking the less likely they were to cease, CPD was positively correlated with smoking cessation (0.13) i.e., heavier smokers were more likely to stop smoking. In contrast, the *DBH* SNV, rs3025343, (first identified via its association with increased smoking cessation [6]) was associated with increased pack-years (*P* = 1.29 × 10^−14^) and increased CPD (*P* = 2.93 × 10^−9^) in our study. The association at *DBH* also represents the first time that a SNV has a smaller *P*-value for pack-years (*n* = 131,892) compared to CPD (*n* = 128,746). These findings may help elucidate the genetic basis of these correlated addiction phenotypes.

We performed the largest exome-wide genetic association study of smoking behaviour-related traits to date and nearly doubled the number of replicated associations to 24 (including conditional analyses) including associations on the X-chromosome for the first time, which merit further study. We also identified a further 35 novel smoking trait associated SNVs in the combined meta-analysis. The novel loci identified in this study will substantially expand our knowledge of the smoking addiction-related traits, facilitate understanding the genetic aetiology of smoking behaviour and may lead to the identification of drug targets of potential relevance to prevent individuals from initiating smoking and/or aid smokers to stop smoking.

## Supplementary Information

Supplementary Material
