# Supplementary material for: Meta-analysis of up to 622,409 individuals identifies 40 novel smoking behaviour associated genetic loci
Source: Mol Psychiatry. 2019 Jan 7;25(10):2392–409. doi: 10.1038/s41380-018-0313-0 (PMC7515840; doi:10.1038/s41380-018-0313-0)
Supplement: Supplementary file 1 — Supplementary Material [file 41380_2018_313_MOESM1_ESM.docx]

Supplementary material:

Meta-analysis of up to 622,409 individuals identifies 40 novel smoking behaviour associated genetic loci

## Acknowledgements and Funding statements

The authors would like to thank the many colleagues who contributed to collection and phenotypic characterisation of the clinical samples, as well as genotyping and analysis of the GWA data. Special mentions are as follows:

**CGSB participating cohorts:** Some of the data utilised in this study were provided by the Understanding Society: The UK Household Longitudinal Study, which is led by the Institute for Social and Economic Research at the University of Essex and funded by the Economic and Social Research Council. The data were collected by NatCen and the genome wide scan data were analysed by the Wellcome Trust Sanger Institute. The Understanding Society DAC have an application system for genetics data and all use of the data should be approved by them. The application form is at: <https://www.understandingsociety.ac.uk/about/health/data>.

The Airwave Health Monitoring Study is funded by the UK Home Office, (Grant number 780-TETRA) with additional support from the National Institute for Health Research Imperial College Health Care NHS Trust and Imperial College Biomedical Research Centre. We thank all participants in the Airwave Health Monitoring Study. This work used computing resources provided by the MRC- funded UK MEDical Bioinformatics partnership programme (UK MED-BIO) (MR/L01632X/1). Paul Elliott wishes to acknowledge the Medical Research Council and Public Health England (MR/L01341X/1) for the MRC-PHE Centre for Environment and Health; and the NIHR Health Protection Research Unit in Health Impact of Environmental Hazards (HPRU-2012-10141). Paul Elliott is supported by the UK Dementia Research Institute which receives its funding from UK DRI Ltd funded by the UK Medical Research Council, Alzheimer’s Society and Alzheimer’s Research UK. Paul Elliott is associate director of the Health Data Research UK London funded by a consortium led by the UK Medical Research Council.

SHIP (Study of Health in Pomerania) and SHIP-TREND both represent population-based studies. SHIP is supported by the German Federal Ministry of Education and Research (Bundesministerium für Bildung und Forschung (BMBF); grants 01ZZ9603, 01ZZ0103, and 01ZZ0403) and the German Research Foundation (Deutsche Forschungsgemeinschaft (DFG); grant GR 1912/5-1). SHIP and SHIP-TREND are part of the Community Medicine Research net (CMR) of the Ernst-Moritz-Arndt University Greifswald (EMAU) which is funded by the BMBF as well as the Ministry for Education, Science and Culture and the Ministry of Labor, Equal Opportunities, and Social Affairs of the Federal State of Mecklenburg-West Pomerania. The CMR encompasses several research projects that share data from SHIP. SNP typing of SHIP and SHIP-TREND using the Illumina Infinium HumanExome BeadChip (version v1.0) was supported by the BMBF (grant 03Z1CN22).

LifeLines authors thank Behrooz Alizadeh, Annemieke Boesjes, Marcel Bruinenberg, Noortje Festen, Ilja Nolte, Lude Franke, Mitra Valimohammadi for their help in creating the GWAS database, and Rob Bieringa, Joost Keers, René Oostergo, Rosalie Visser, Judith Vonk for their work related to data-collection and validation. The authors are grateful to the study participants, the staff from the LifeLines Cohort Study and Medical Biobank Northern Netherlands, and the participating general practitioners and pharmacists. LifeLines Scientific Protocol Preparation: Rudolf de Boer, Hans Hillege, Melanie van der Klauw, Gerjan Navis, Hans Ormel, Dirkje Postma, Judith Rosmalen, Joris Slaets, Ronald Stolk, Bruce Wolffenbuttel; LifeLines GWAS Working Group: Behrooz Alizadeh, Marike Boezen, Marcel Bruinenberg, Noortje Festen, Lude Franke, Pim van der Harst, Gerjan Navis, Dirkje Postma, Harold Snieder, Cisca Wijmenga, Bruce Wolffenbuttel. The authors wish to acknowledge the services of the LifeLines Cohort Study, the contributing research centres delivering data to LifeLines, and all the study participants. Niek Verweij was supported by NWO VENI (016.186.125).

Fenland authors thank Fenland Study volunteers for their time and help, Fenland Study general Practitioners and practice staff for assistance with recruitment, and Fenland Study Investigators, Co-ordination team and the Epidemiology Field, Data and Laboratory teams for study design, sample/data collection and genotyping.

We thank all ASCOT trial participants, physicians, nurses, and practices in the participating countries for their important contribution to the study. In particular we thank Clare Muckian and David Toomey for their help in DNA extraction, storage, and handling. We would also like to acknowledge the Barts and The London Genome Centre staff for genotyping the Exome Chip array.

The BRIGHT study is extremely grateful to all the patients who participated in the study and the BRIGHT nursing team. We would also like to thank the Barts Genome Centre staff for their assistance with this project.

Patricia B. Munroe, Mark J. Caulfield, and Helen R. Warren wish to acknowledge the NIHR Cardiovascular Biomedical Research Unit at Barts and The London, Queen Mary University of London, UK for support. Mark J. Caulfield are Senior National Institute for Health Research Investigators.

EMBRACE Collaborating Centres are: Coordinating Centre, Cambridge: Daniel Barrowdale, Debra Frost, Jo Perkins. North of Scotland Regional Genetics Service, Aberdeen: Zosia Miedzybrodzka, Helen Gregory. Northern Ireland Regional Genetics Service, Belfast: Patrick Morrison, Lisa Jeffers. West Midlands Regional Clinical Genetics Service, Birmingham: Kai-ren Ong, Jonathan Hoffman. South West Regional Genetics Service, Bristol: Alan Donaldson, Margaret James. East Anglian Regional Genetics Service, Cambridge: Joan Paterson, Marc Tischkowitz, Sarah Downing, Amy Taylor. Medical Genetics Services for Wales, Cardiff: Alexandra Murray, Mark T. Rogers, Emma McCann. St James’s Hospital, Dublin & National Centre for Medical Genetics, Dublin: M. John Kennedy, David Barton. South East of Scotland Regional Genetics Service, Edinburgh: Mary Porteous, Sarah Drummond. Peninsula Clinical Genetics Service, Exeter: Carole Brewer, Emma Kivuva, Anne Searle, Selina Goodman, Kathryn Hill. West of Scotland Regional Genetics Service, Glasgow: Rosemarie Davidson, Victoria Murday, Nicola Bradshaw, Lesley Snadden, Mark Longmuir, Catherine Watt, Sarah Gibson, Eshika Haque, Ed Tobias, Alexis Duncan. South East Thames Regional Genetics Service, Guy’s Hospital London: Louise Izatt, Chris Jacobs, Caroline Langman. North West Thames Regional Genetics Service, Harrow: Huw Dorkins. Leicestershire Clinical Genetics Service, Leicester: Julian Barwell. Yorkshire Regional Genetics Service, Leeds: Julian Adlard, Gemma Serra-Feliu. Cheshire & Merseyside Clinical Genetics Service, Liverpool: Ian Ellis, Claire Foo. Manchester Regional Genetics Service, Manchester: D Gareth Evans, Fiona Lalloo, Jane Taylor. North East Thames Regional Genetics Service, NE Thames, London: Lucy Side, Alison Male, Cheryl Berlin. Nottingham Centre for Medical Genetics, Nottingham: Jacqueline Eason, Rebecca Collier. Northern Clinical Genetics Service, Newcastle: Alex Henderson, Oonagh Claber, Irene Jobson. Oxford Regional Genetics Service, Oxford: Lisa Walker, Diane McLeod, Dorothy Halliday, Sarah Durell, Barbara Stayner. The Institute of Cancer Research and Royal Marsden NHS Foundation Trust: Ros Eeles, Nazneen Rahman, Elizabeth Bancroft, Elizabeth Page, Audrey Ardern-Jones, Kelly Kohut, Jennifer Wiggins, Jenny Pope, Sibel Saya, Natalie Taylor, Zoe Kemp and Angela George. North Trent Clinical Genetics Service, Sheffield: Jackie Cook, Oliver Quarrell, Cathryn Bardsley. South West Thames Regional Genetics Service, London: Shirley Hodgson, Sheila Goff, Glen Brice, Lizzie Winchester, Charlotte Eddy, Vishakha Tripathi, Virginia Attard. Wessex Clinical Genetics Service, Princess Anne Hospital, Southampton: Diana Eccles, Anneke Lucassen, Gillian Crawford, Donna McBride, Sarah Smalley.

Understanding Society Scientific Group is funded by the Economic and Social Research Council (ES/H029745/1) and the Wellcome Trust (WT098051). Paul D.P. Pharoah is funded by Cancer Research UK (C490/A16561). SHIP is funded by the German Federal Ministry of Education and Research (BMBF) and the German Research Foundation (DFG); see acknowledgements for details. F.W. Asselbergs is funded by the Netherlands Heart Foundation (2014T001) and supported by UCL Hospitals NIHR Biomedical Research Centre. The LifeLines Cohort Study, and generation and management of GWAS genotype data for the LifeLines Cohort Study is supported by the Netherlands Organization of Scientific Research NWO (grant 175.010.2007.006), the Economic Structure Enhancing Fund (FES) of the Dutch government, the Ministry of Economic Affairs, the Ministry of Education, Culture and Science, the Ministry for Health, Welfare and Sports, the Northern Netherlands Collaboration of Provinces (SNN), the Province of Groningen, University Medical Center Groningen, the University of Groningen, Dutch Kidney Foundation and Dutch Diabetes Research Foundation. Niek Verweij is supported by Horizon 2020, Marie Sklodowska-Curie (661395) and ICIN-NHI.

Phenotype collection in the Lothian Birth Cohort 1921 was supported by the UK’s Biotechnology and Biological Sciences Research Council (BBSRC), The Royal Society and The Chief Scientist Office of the Scottish Government. Phenotype collection in the Lothian Birth Cohort 1936 was supported by Age UK (The Disconnected Mind project). Genotyping was supported by Centre for Cognitive Ageing and Cognitive Epidemiology (Pilot Fund award), Age UK, and the Royal Society of Edinburgh. The work was undertaken by The University of Edinburgh Centre for Cognitive Ageing and Cognitive Epidemiology, part of the cross council Lifelong Health and Wellbeing Initiative (MR/K026992/1). Funding from the BBSRC and Medical Research Council (MRC) is gratefully acknowledged. Paul W. Franks is supported by Novo Nordisk, the Swedish Research Council, Påhlssons Foundation, Swedish Heart Lung Foundation (2020389), and Skåne Regional Health Authority. Nicholas J Wareham, Claudia Langenberg, Robert A Sacott, and Jian'an Luan are supported by the MRC (MC_U106179471 and MC_UU_12015/1). The BRIGHT study was supported by the Medical Research Council of Great Britain (Grant Number G9521010D); and by the British Heart Foundation (Grant Number PG/02/128). The BRIGHT study is extremely grateful to all the patients who participated in the study and the BRIGHT nursing team. The Exome Chip genotyping was funded by Wellcome Trust Strategic Awards (083948 and 085475). We would also like to thank the Barts Genome Centre staff for their assistance with this project. The ASCOT study and the collection of the ASCOT DNA repository was supported by Pfizer, New York, NY, USA, Servier Research Group, Paris, France; and by Leo Laboratories, Copenhagen, Denmark. Genotyping of the Exome Chip in ASCOT-SC and ASCOT-UK was funded by the National Institutes of Health Research (NIHR). Anna F. Dominiczak was supported by the British Heart Foundation (Grant Numbers RG/07/005/23633, SP/08/005/25115); and by the European Union Ingenious HyperCare Consortium: Integrated Genomics, Clinical Research, and Care in Hypertension (grant number LSHM-C7-2006-037093). Nilesh J. Samani is supported by the British Heart Foundation and is a Senior National Institute for Health Research Investigator. Panos Deloukas is supported by the British Heart Foundation (RG/14/5/30893), and NIHR, where his work forms part of the research themes contributing to the translational research portfolio of Barts Cardiovascular Biomedical Research Centre which is funded by the National Institute for Health Research (NIHR).

Generation Scotland received core support from the Chief Scientist Office of the Scottish Government Health Directorates [CZD/16/6] and the Scottish Funding Council [HR03006]. Genotyping of the GS:SFHS samples was carried out by the Genetics Core Laboratory at the Clinical Research Facility, University of Edinburgh, Scotland and was funded by the Medical Research Council UK and the Wellcome Trust (Wellcome Trust Strategic Award “STratifying Resilience and Depression Longitudinally” (STRADL) Reference 104036/Z/14/Z).

We would like to acknowledge the contributions of the recruitment team in Korcula, the administrative teams in Croatia and Edinburgh and the people of Korcula. Exome array genotyping was performed at the Clinical Research Facility University of Edinburgh, Scotland and funded by the Medical Research Council UK.

The LOLIPOP study is supported by the National Institute for Health Research (NIHR) Comprehensive Biomedical Research Centre Imperial College Healthcare NHS Trust, the British Heart Foundation (SP/04/002), the Medical Research Council (G0601966, G0700931), the Wellcome Trust (084723/Z/08/Z, 090532 & 098381) the NIHR (RP-PG-0407-10371), the NIHR Official Development Assistance (ODA, award 16/136/68), the European Union FP7 (EpiMigrant, 279143) and H2020 programs (iHealth-T2D, 643774). We acknowledge support of the MRC-PHE Centre for Environment and Health, and the NIHR Health Protection Research Unit on Health Impact of Environmental Hazards. The work was carried out in part at the NIHR/Wellcome Trust Imperial Clinical Research Facility. The views expressed are those of the author(s) and not necessarily those of the Imperial College Healthcare NHS Trust, the NHS, the NIHR or the Department of Health. We thank the participants and research staff who made the study possible. JC is supported by the Singapore Ministry of Health’s National Medical Research Council under its Singapore Translational Research Investigator (STaR) Award (NMRC/STaR/0028/2017).

The research was supported by the National Institute for Health Research (NIHR) Exeter Clinical Research Facility and ERC grant 323195; SZ-245 50371-GLUCOSEGENES-FP7-IDEAS-ERC to T.M. Frayling. Hanieh Yaghootkar is funded by Diabetes UK RD Lawrence fellowship (grant:17/0005594)

Anna Dominiczak was funded by a BHF Centre of Research Excellence Award (RE/13/5/30177)

**GSCAN participating cohorts:**

The Collaborative Study on the Genetics of Alcoholism (COGA), Principal Investigators: B. Porjesz, V. Hesselbrock, H. Edenberg, L. Bierut. The study includes eleven different centers: University of Connecticut (V. Hesselbrock); Indiana University (H.J. Edenberg, J. Nurnberger Jr., T. Foroud); University of Iowa (S. Kuperman, J. Kramer); SUNY Downstate (B. Porjesz); Washington University in St. Louis (L. Bierut, J. Rice, K. Bucholz, A. Agrawal); University of California at San Diego (M. Schuckit); Rutgers University (J. Tischfield, A. Brooks); Department of Biomedical and Health Informatics, The Children’s Hospital of Philadelphia; Department of Genetics, Perelman School of Medicine, University of Pennsylvania, Philadelphia PA (L. Almasy), Virginia Commonwealth University (D. Dick), Icahn School of Medicine at Mount Sinai (A. Goate), and Howard University (R. Taylor). Other COGA collaborators include: L. Bauer (University of Connecticut); J. McClintick, L. Wetherill, X. Xuei, Y. Liu, D. Lai, S. O’Connor, M. Plawecki, S. Lourens (Indiana University); G. Chan (University of Iowa; University of Connecticut); J. Meyers, D. Chorlian, C. Kamarajan, A. Pandey, J. Zhang (SUNY Downstate); J.-C. Wang, M. Kapoor, S. Bertelsen (Icahn School of Medicine at Mount Sinai); A. Anokhin, V. McCutcheon, S. Saccone (Washington University); J. Salvatore, F. Aliev, B. Cho (Virginia Commonwealth University); and Mark Kos (University of Texas Rio Grande Valley). A. Parsian and M. Reilly are the NIAAA Staff Collaborators. COGA investigators continue to be inspired by their memories of Henri Begleiter and Theodore Reich, founding PI and Co-PI of COGA, and also owe a debt of gratitude to other past organizers of COGA, including Ting-Kai Li, P. Michael Conneally, Raymond Crowe, and Wendy Reich, for their critical contributions. COGA investigators are very grateful to Dr. Bruno Buecher without whom this project would not have existed.

The authors also thank all those at the GECCO Coordinating Center for helping bring together the data and people that made this project possible. ASTERISK, a GECCO sub-study, also thanks all those who agreed to participate in this study, including the patients and the healthy control persons, as well as all the physicians, technicians and students. As part of the GECCO sub-studies, CPS-II authors thank the CPS-II participants and Study Management Group for their invaluable contributions to this research. The authors would also like to acknowledge the contribution to this study from central cancer registries supported through the Centers for Disease Control and Prevention National Program of Cancer Registries, and cancer registries supported by the National Cancer Institute Surveillance Epidemiology and End Results program. Another GECCO sub-study, HPFS and NHS investigators would like to acknowledge Patrice Soule and Hardeep Ranu of the Dana Farber Harvard Cancer Center High-Throughput Polymorphism Core who assisted in the genotyping for NHS, HPFS under the supervision of Dr. Immaculata Devivo and Dr. David Hunter, Qin (Carolyn) Guo and Lixue Zhu who assisted in programming for NHS and HPFS. HPFS and NHS investigators also thank the participants and staff of the Nurses' Health Study and the Health Professionals Follow-Up Study, for their valuable contributions as well as the following state cancer registries for their help: AL, AZ, AR, CA, CO, CT, DE, FL, GA, ID, IL, IN, IA, KY, LA, ME, MD, MA, MI, NE, NH, NJ, NY, NC, ND, OH, OK, OR, PA, RI, SC, TN, TX, VA, WA, WY. The authors assume full responsibility for analyses and interpretation of these data. PLCO, a substudy within GECCO, was supported by the Intramural Research Program of the Division of Cancer Epidemiology and Genetics, and additionally supported by contracts from the Division of Cancer Prevention, National Cancer Institute, NIH, DHHS. Additionally, a subset of control samples were genotyped as part of the Cancer Genetic Markers of Susceptibility (CGEMS) Prostate Cancer GWAS^1^, CGEMS pancreatic cancer scan (PanScan)^2, 3^, and the Lung Cancer and Smoking study^4^. The prostate and PanScan study datasets were accessed with appropriate approval through the dbGaP online resource (<http://cgems.cancer.gov/data/>) accession numbers phs000207.v1.p1 and phs000206.v3.p2, respectively, and the lung datasets were accessed from the dbGaP website (<http://www.ncbi.nlm.nih.gov/gap>) through accession number phs000093.v2.p2. For the lung study, the GENEVA Coordinating Center provided assistance with genotype cleaning and general study coordination, and the Johns Hopkins University Center for Inherited Disease Research conducted genotyping. The authors thank Drs. Christine Berg and Philip Prorok, Division of Cancer Prevention, National Cancer Institute, the Screening Center investigators and staff or the Prostate, Lung, Colorectal, and Ovarian (PLCO) Cancer Screening Trial, Mr. Tom Riley and staff, Information Management Services, Inc., Ms. Barbara O’Brien and staff, Westat, Inc., and Drs. Bill Kopp and staff, SAIC-Frederick. Most importantly, we acknowledge the study participants for their contributions to making this study possible.

We also thank all participants and staff of the André and France Desmarais Montreal Heart Institute’s (MHI) Biobank. The genotyping of the MHI Biobank was done at the MHI Pharmacogenomic Centre and funded by the MHI Foundation.

HRS is supported by the National Institute on Aging (NIA U01AG009740). The genotyping was funded separately by the National Institute on Aging (RC2 AG036495, RC4 AG039029). Our genotyping was conducted by the NIH Center for Inherited Disease Research (CIDR) at Johns Hopkins University. Genotyping quality control and final preparation of the data were performed by the University of Michigan School of Public Health.

**CHDExome+ participating cohorts:**

**BRAVE:** The BRAVE study genetic epidemiology working group is a collaboration between the Cardiovascular Epidemiology Unit, Department of Public Health and Primary Care, University of Cambridge, UK, the Centre for Control of Chronic Diseases, icddr,b, Dhaka, Bangladesh and the National Institute of Cardiovascular Diseases, Dhaka, Bangladesh.

**CCHS, CIHDS, and CGPS** collaborators thank participants and staff of the Copenhagen City Heart Study, Copenhagen Ischemic Heart Disease Study, and the Copenhagen General Population Study for their important contributions.

**EPIC-CVD:** CHD case ascertainment and validation, genotyping, and clinical chemistry assays in EPIC-CVD were principally supported by grants awarded to the University of Cambridge from the EU Framework Programme 7 (HEALTH-F2-2012-279233), the UK Medical Research Council (G0800270 and MR/L003120/1) and British Heart Foundation (SP/09/002 and RG/13/13/30194), and the European Research Council (268834). We thank all EPIC participants and staff for their contribution to the study, the laboratory teams at the Medical Research Council Epidemiology Unit for sample management and Cambridge Genomic Services for genotyping, Sarah Spackman for data management, and the team at the EPIC-CVD Coordinating Centre for study coordination and administration.

**MORGAM:** The work by MORGAM collaborators has been sustained by the MORGAM Project's recent funding: European Union FP 7 projects ENGAGE (HEALTH-F4-2007-201413), CHANCES (HEALTH-F3-2010-242244) and BiomarCaRE (278913). This has supported central coordination, workshops and part of the activities of the The MORGAM Data Centre, at THL in Helsinki, Finland. MORGAM Participating Centres are funded by regional and national governments, research councils, charities, and other local sources. **PROSPER**: collaborators have received funding from the European Union's Seventh Framework Programme (FP7/2007-2013) under grant agreement n° HEALTH-F2-2009-223004

**PROMIS:** The PROMIS collaborators are are thankful to all the study participants in Pakistan. Recruitment in PROMIS was funded through grants available to investigators at the Center for Non-Communicable Diseases, Pakistan (Danish Saleheen and Philippe Frossard) and investigators at the University of Cambridge, UK (Danish Saleheen and John Danesh). Field-work, genotyping, and standard clinical chemistry assays in PROMIS were principally supported by grants awarded to the University of Cambridge from the British Heart Foundation, UK Medical Research Council, Wellcome Trust, EU Framework 6-funded Bloodomics Integrated Project, Pfizer. We would like to acknowledge the contributions made by the following individuals who were involved in the field work and other administrative aspects of the study: Mohammad Zeeshan Ozair, Usman Ahmed, Abdul Hakeem, Hamza Khalid, Kamran Shahid, Fahad Shuja, Ali Kazmi, Mustafa Qadir Hameed, Naeem Khan, Sadiq Khan, Ayaz Ali, Madad Ali, Saeed Ahmed, Muhammad Waqar Khan, Muhammad Razaq Khan, Abdul Ghafoor, Mir Alam, Riazuddin, Muhammad Irshad Javed, Abdul Ghaffar, Tanveer Baig Mirza, Muhammad Shahid, Jabir Furqan, Muhammad Iqbal Abbasi, Tanveer Abbas, Rana Zulfiqar, Muhammad Wajid, Irfan Ali, Muhammad Ikhlaq, Danish Sheikh and Muhammad Imran.

**INTERVAL:**

Participants in the INTERVAL randomised controlled trial were recruited with the active collaboration of NHS Blood and Transplant England ([www.nhsbt.nhs.uk](http://www.nhsbt.nhs.uk)), which has supported field work and other elements of the trial. DNA extraction and genotyping was funded by the National Institute of Health Research (NIHR), the NIHR BioResource (<http://bioresource.nihr.ac.uk/>) and the NIHR Cambridge Biomedical Research Centre at the Cambridge University Hospitals NHS Foundation Trust ([www.cambridge-brc.org.uk](http://www.cambridge-brc.org.uk)). The academic coordinating centre for INTERVAL was supported by core funding from: NIHR Blood and Transplant Research Unit in Donor Health and Genomics (NIHR BTRU-2014-10024), UK Medical Research Council (MR/L003120/1), British Heart Foundation (RG/13/13/30194), and NIHR Research Cambridge Biomedical Research Centre. A complete list of the investigators and contributors to the INTERVAL trial is provided in reference^5^.

The views expressed are those of the authors and not necessarily those of the NHS, the NIHR or the Department of Health and Social Care.

## Consortia membership

**Understanding Society**: The UK Household Longitudinal Study: Michaela Benzeval^a^, Jonathan Burton^a^, Nicholas Buck^a^, Annette Jäckle^a^, Meena Kumari^a^, Heather Laurie^a^, Peter Lynn^a^, Stephen Pudney^a^, Birgitta Rabe^a^, Dieter Wolke^b^.

1. Institute for Social and Economic Research, University of Essex, UK
2. University of Warwick, UK

**EPIC-CVD consortium**

Kim Overvad^1,2^, Anne Tjønneland^3^, Francoise Clavel-Chapelon^4^, Rudolf Kaaks^5^, Heiner Boeing^6^, Antonia Trichopoulou^7,8^, Pietro Ferrari^9^, Domenico Palli^10^, Vittorio Krogh^11^, Salvatore Panico^12^, Rosario Tumino^13^, Giuseppe Matullo^14,15^, Jolanda Boer^16^, Yvonne van. der Schouw^17^, Elisabete Weiderpass^18,19,20,21^, J. Ramon Quiros^22^, María-José Sánchez^23,24^, Carmen Navarro^25^, Conchi Moreno-Iribas^26^, Larraitz Arriola^27^, Olle Melander^28^, Patrik Wennberg^29^, Nicholas J. Wareham^30^, Timothy J. Key^31^, Elio Riboli^32^, Adam S. Butterworth^33,34^, Joanna M M Howson^33^, John Danesh^33,34,35^

1. Department of Public Health, Section for Epidemiology, Aarhus University, Aarhus, Denmark
2. Department of Cardiology, Aalborg University Hospital, Aalborg, Denmark
3. Diet, Genes and Environment, Danish Cancer Society Research Center, Copenhagen, Denmark
4. INSERM, Centre for Research in Epidemiology and Population Health (CESP), U1018, Nutrition, Hormones, and Women's Health Team, Institut Gustave Roussy, Villejuif, France
5. Division of Cancer Genetic Epidemiology, German Cancer Research Centre (DKFZ), im Neuenheimer Feld 581, 69120 Heidelberg, Germany
6. Department of Epidemiology, German Institute of Human Nutrition (DIfE), Potsdam-Rehbrücke, Germany
7. WHO Collaborating Center for Nutrition and Health, Unit of Nutritional Epidemiology and Nutrition in Public Health, Department of Hygiene, Epidemiology and Medical Statistics, University of Athens Medical School, Athens, Greece
8. Hellenic Health Foundation, Athens, Greece
9. International Agency for Research on Cancer, Lyon, France
10. Molecular and Nutritional Epidemiology Unit, Centro per lo Studio e la Prevenzione Oncologica-Scientific Institute of Tuscany, Florence, Italy
11. Epidemiology and Prevention Unit, Fondazione IRCCS Istituto Nazionale dei Tumori, Milan, Italy
12. Dipartimento di Medicina Clinica e Chirurgia, Federico II University, Naples, Italy
13. Cancer Registry and Histopathology Unit, Civic- M.P.Arezzo Hospital, ASP Ragusa, Italy
14. Human Genetics Foundation, Turin, Italy
15. Department of Medical Sciences, University of Turin, Italy
16. Centre for Nutrition, Prevention and Health Services, National Institute for Public Health and the Environment (RIVM), Bilthoven, the Netherlands
17. Julius Center for Health Sciences and Primary Care, University Medical Center Utrecht, Utrecht, the Netherlands
18. Department of Community Medicine, Faculty of Health Sciences, University of Tromsø, The Arctic University of Norway, Tromsø, Norway
19. Department of Research, Cancer Registry of Norway, Institute of Population-Based Cancer Research, Oslo, Norway
20. Department of Medical Epidemiology and Biostatistics, Karolinska Institutet, Stockholm, Sweden
21. Genetic Epidemiology Group, Folkhälsan Research Center, Helsinki, Finland
22. Public Health Directorate, Asturias, Spain
23. Escuela Andaluza de Salud Pública, Instituto de Investigación Biosanitaria ibs.GRANADA. Hospitales Universitarios de Granada/Universidad de Granada, Granada, Spain
24. CIBER de Epidemiología y Salud Pública (CIBERESP), Madrid, Spain

25. Epidemiology Department, Murcia Health Authority, Murcia, Spain
26. Public Health Institute of Navarra, Pamplona, Spain
27. Public Health Division of Gipuzkoa, Instituto Bio-Donostia, Basque Government, CIBERESP, Spain
28. Department of Clinical Sciences, Hypertension & Cardiovascular Disease, Clinical Research Centre, Malmö University Hospital, Malmö, Sweden
29. Department of Public Health and Clinical Medicine, Family Medicine, Umeå University, Umeå, Sweden
30. Medical Research Council Epidemiology Unit, University of Cambridge, Cambridge, UK
31. Cancer Epidemiology Unit, Nuffield Department of Population Health, University of Oxford, UK
32. School of Public Health, Imperial College London, UK
33. MRC/BHF Cardiovascular Epidemiology Unit, Department of Public Health & Primary Care, University of Cambridge, UK
34. The National Institute for Health Research Blood and Transplant Unit (NIHR BTRU) in Donor Health and Genomics at the University of Cambridge, UK
35. Wellcome Trust Sanger Institute, Genome Campus, Hinxton, UK

## Figures

**Supp. Figures 1a-d:** Manhattan plots of all four smoking behaviour related trait association studies (at discovery stage). Plots are shown of genome-wide association results for Smoking cessation (top), Cigarettes per day, Pack-years, and Smoking initiation (bottom). Previously reported signals are shown in dark blue, and new signals are shown in red. Signals are shown only for the trait with which they exhibited the strongest association. The red and blue lines correspond to the genome-wide significance level (*P*=5×10^−8^; –log_10_*P*=7.3) and suggestive significance (*P*=5×10^−7^; –log_10_*P*=6.3), respectively. Labels are for the nearest gene to the new sentinel variants. The top signals were truncated at 10^-14^ for clarity. The image was created using a modified version of the R package qqman. NB: SNVs in/near *REV3L*, *CNNM2* and *TMEM182* replicated in the replication stage (for SI).


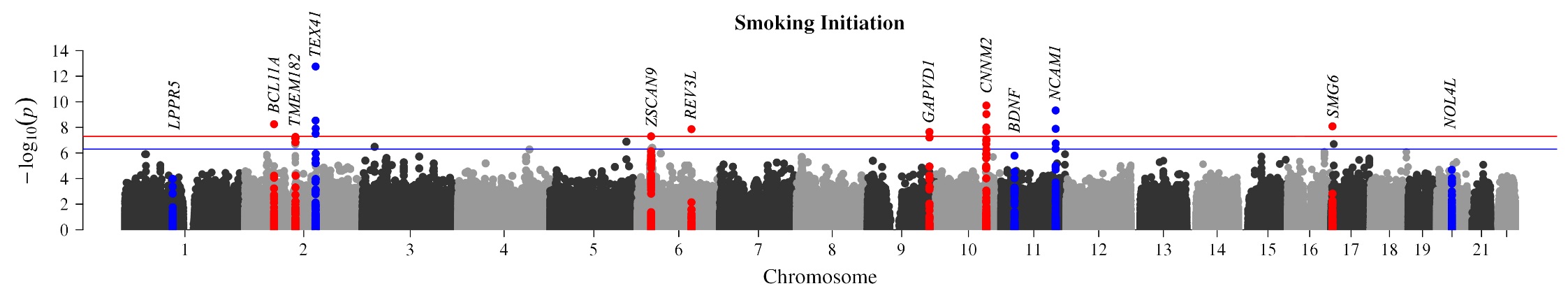


**
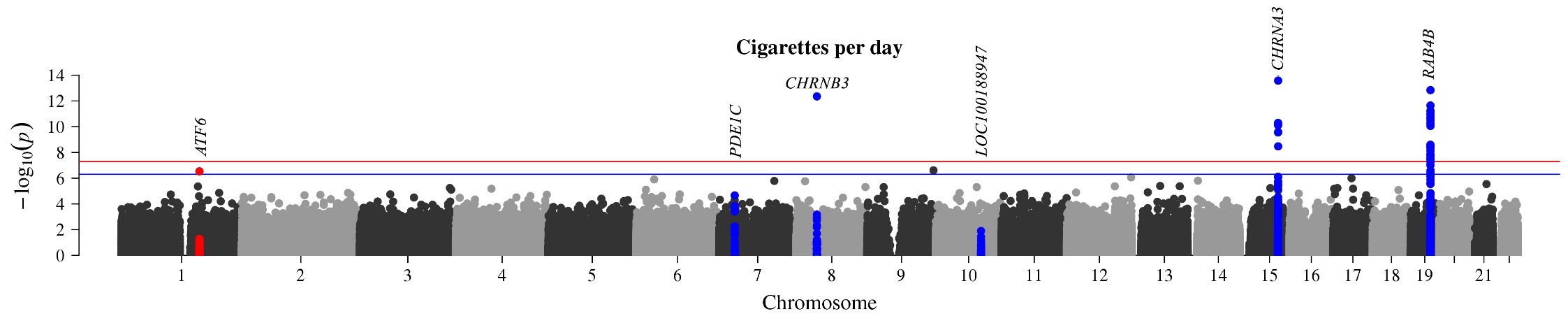
**

**
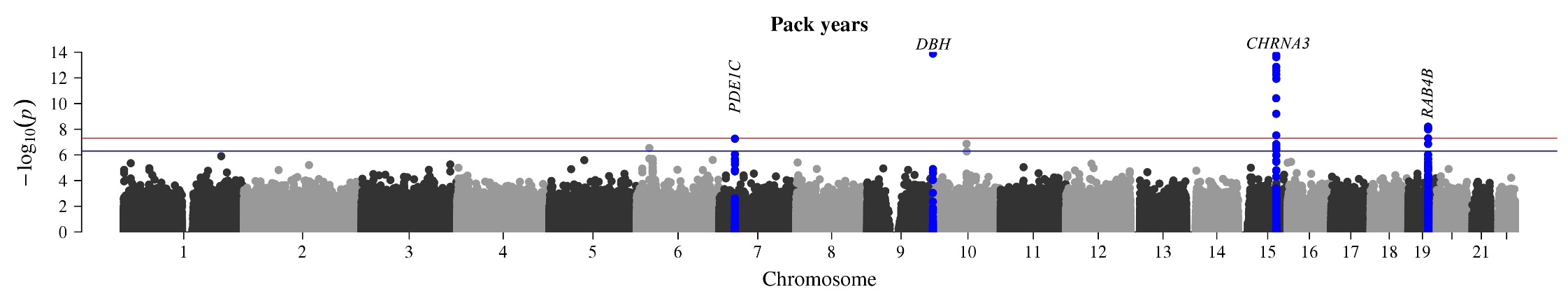
**

**
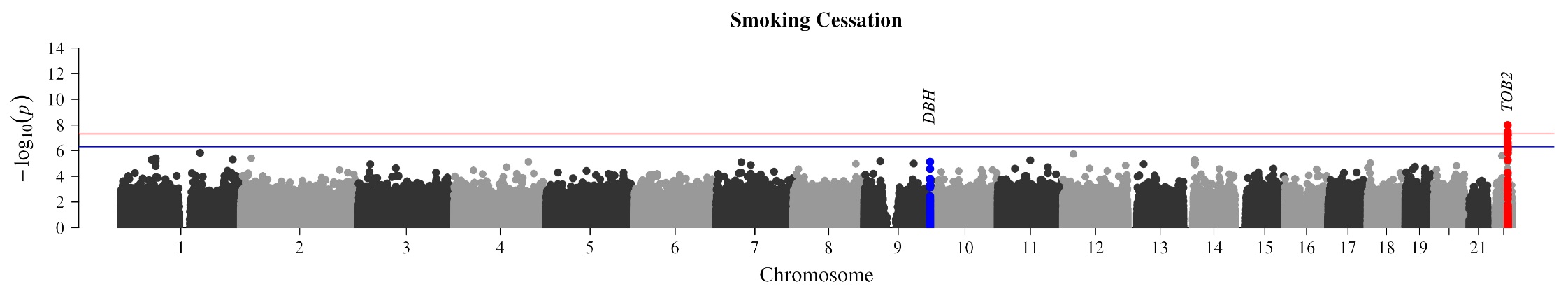
**

**Supp. Figures 2a-d:** Quantile-Quantile (QQ)-plots and genomic inflation factor (λ) for Smoking Initiation (SI), Smoking Cessation (SC), Cigarettes per day (CPD), and Pack-years (PY) meta-analyses (discovery stage).


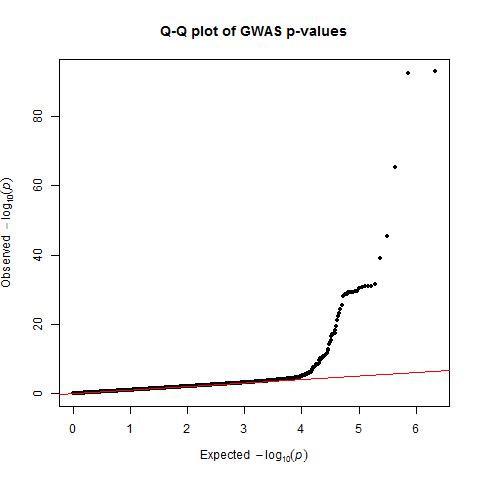

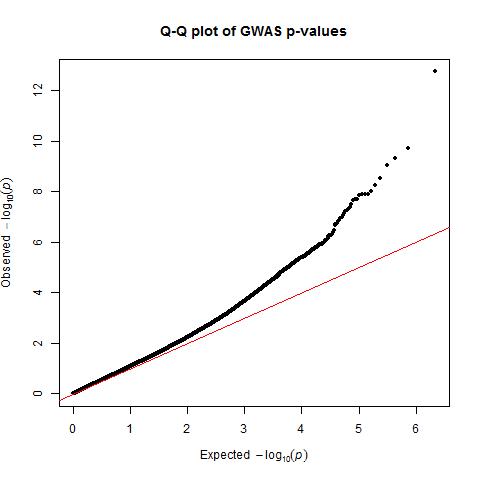

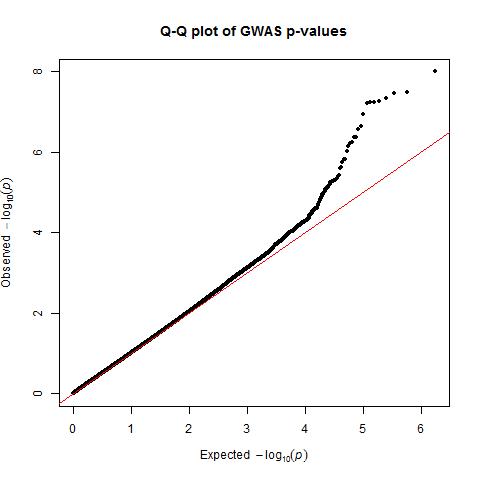


λ= 1.04

**SI**

**SC**

**CPD**

λ= 1.02

λ= 1.11


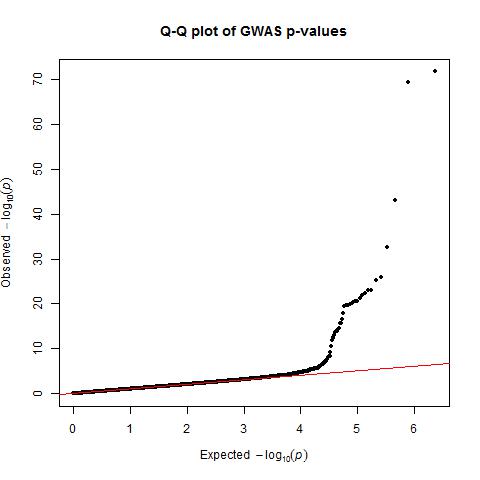


**PY**

λ= 1.02

## Tables

**Supp. Table 1:** Studies which contributed to primary analyses (at discovery stage), the consortia name, and sample size, gender distribution and ancestry of each dataset. CGSB: Consortium for the Genetics of Smoking Behaviour (Leicester); CHDExome+: Coronary Heart Disease Exome+ consortium (Cambridge); GSCAN: GWAS & Sequencing Consortium of Alcohol and Nicotine (Colorado & Michigan). Affiliation with same consortia implies that a similar study-level QC protocol and analysis plan was followed. DNC: Did not contribute or excluded due to quality control issues; n: sample size; N/A: information not available.

|  | **Cohort/Sample collections** | **Consortia** | **Smoking initiation n (smokers/non-smokers)** | **Cigarettes per day n (mean/sd)** | **Pack-years n (mean/sd)** | **Smoking cessation n (current smokers/ex-smokers)** | **Gender distribution for Smoking initiation samples (Male/Female)** | **Ancestry of samples** |
| --- | --- | --- | --- | --- | --- | --- | --- | --- |
| 1 | Airwave | CGSB | 1905 (556/1349) | 160 (9.5/6.5) | 556 (10.2/9.5) | 556 (396/160) | 1210/695 | White European |
| 2 | ASCOT – Scotland dataset | CGSB | 2461 (1737/724) | 1029 (12.9/7.9) | DNC | 1738 (688/1070) | 1833/629 | White European |
| 3 | ASCOT – UK dataset | CGSB | 3243 (2267/976) | 725 (13.2/9.6) | DNC | 2267 (1462/805) | 2659/587 | White European |
| 4 | 1958BC | CGSB | 5537 (2943/2594) | 2839 (18.72/10.21) | 2258 (16.93/10.7) | 2738 (1078/1660) | 3264/2553 | White European |
| 5 | BRIGHT | CGSB | 851 (401/450) | 376 (17.2/11.2) | 360 (23.5/19.8) | 401 (287/114) | 508/851 | White European |
| 6 | DIABNORD | CGSB | 397 (175/222) | DNC | DNC | 175 (88/87) | 193/204 | White European |
| 7 | EFSOCH | CGSB | 1389 (689/700) | 385 (10.32/9.9) | DNC | 208 (100/108) | 701/688 | White European |
| 8 | EGCUT – BMI dataset | CGSB | 929 (506/423) | DNC | 500 (15.7/14.6) | DNC | 464/465 | White European |
| 9 | EGCUT – Controls dataset | CGSB | 807 (304/529) | 293 (13.42/8.86) | 292 (12.82/13.75) | 294 (149/155) | 407/400 | White European |
| 10 | EGCUT - Height cases dataset | CGSB | DNC | 429 (12.91/7.68) | 421 (12.07/13.72) | 432 (129/306) | N/A | White European |
| 11 | EGCUT – Psoriasis cases dataset | CGSB | DNC | 409 (12.19/8.11) | 407 (10.08/11.54) | 414 (257/157) | N/A | White European |
| 12 | EGCUT - T2D cases dataset | CGSB | 836 (347/507) | DNC | DNC | 337 (195/152) | 366/470 | White European |
| 13 | EGCUT – CoreExome dataset | CGSB | 4642 (1955/2687) | 1904 (10.67/7.29) | 1884 (6.22/7.63) | 1955 (503/1452) | 1518/3124 | White European |
| 14 | EMBRACE | CGSB | 604 (296/308) | 290 (11.72/7.17) | 286 (12.47/11.61) | 295 (198/97) | 0/604 | White European |
| 15 | Fenland | CGSB | 1333 (632/701) | 425 (11.4/9.1) | 290 (12.7/13.0) | 632 (443/189) | 619/714 | White European |
| 16 | FIA3 | CGSB | 2387 (1429/958) | DNC | DNC | 1429 (491/938) | 1612/775 | White European |
| 17 | GS:SFHS | CGSB | 9810 (4705/5105) | 2511 (14.19/9.64) | 4824 (18.07/18.76) | 4470 (2916/1554) | 5760/4050 | White European |
| 18 | GLACIER | CGSB | 928 (432/496) | DNC | DNC | 432 (226/206) | 420/508 | White European |
| 19 | GoDARTS | CGSB | 4447 (2746/1701) | 2578 (7.72/5.1) | 2575 (18.45/13.33) | 2745 (720/2025) | 2673/1774 | White European |
| 20 | KORA F4 | CGSB | 2843 (1664/1179) | 443 (15.24/8.75) | 1591 (29.81/21.33) | 1680 (1155/525) | 1378/1465 | White European |
| 21 | CROATIA-Korcula | CGSB | 836 (430/406) | 415 (19.42/14.55) | 415 (21.20/26.04) | 410 (222/195) | 523/313 | White European |
| 22 | LBC1921 | CGSB | 503 (284/219) | 283 (15.43/11.02) | 280 (28.14/24.37) | 284 (247/37) | 208/295 | White European |
| 23 | LBC1936 | CGSB | 983 (527/456) | 518 (17.61/12.61) | 516 (31.04/27.37) | 527 (421/106) | 498/485 | White European |
| 24 | LifeLines | CGSB | DNC | 1012 (11.35/9.54) | 1036 (13.7/11.34) | 1058 (578/480) | N/A | White European |
| 25 | LOLIPOP Exome chip dataset | CGSB | 1664 (301/1363) | 1663 (11.5/8.78) | 1636 (14.94/13.85) | 301 (157/144) | 1241/423 | White European |
| 26 | LOLIPOP OmniExpress chip dataset | CGSB | 977 (158/819) | 975 (10.45/10.34) | 961 (12.41/13.81) | 158 (73/85) | 560/417 | White European |
| 27 | LRGP | CGSB | 2070 (1620/441) | 389 (14.42/8.11) | 987 (16.91/10.27) | 2061 (668/952) | 1065/1335 | White European |
| 28 | OxBB | CGSB | 4301 (1701/2600) | 1698 (11.6/9.01) | 1652 (15.3/14.78) | 1719 (1229/490) | 2010/2291 | White European |
| 29 | SEARCH – Breast Cancer dataset | CGSB | 3465 (1722/1743) | 534 (13.65/7.33) | 1616 (16.71/12.77) | 1722 (757/965) | 0/3465 | White European |
| 30 | SEARCH – Controls dataset | CGSB | 1810 (839/971) | 206 (12.89/7.33) | 777 (18.66/15.46) | 839 (598/241) | 958/852 | White European |
| 31 | SEARCH – Ovarian Cancer dataset | CGSB | 723 (298/425) | 56 (14.52/6.97) | 270 (17.65/14.78) | 298 (204/94) | 0/723 | White European |
| 32 | SHIP | CGSB | 7396 (3484/3912) | 1875 (14.49/7.66) | 3465 (18.81/15.92) | 3484 (1609/1875) | 3573/3823 | White European |
| 33 | SIBS | CGSB | 878 (392/486) | 375 (12.7/8.04) | 375 (16.04/6.18) | 392 (306/86) | 0/878 | White European |
| 34 | UKHLS | CGSB | 9176 (5111/4185) | 1712 (13.75/8.16) | 2683 (20.23/21.63) | 5111 (3338/1773) | 4086/5210 | White European |
| 35 | ARIC | GSCAN | 8970 | 5381 | 5304 | DNC | N/A | White European |
| 36 | COGA | GSCAN | DNC | 1465 | 1435 | DNC | N/A | White European |
| - | COGA - replication | GSCAN | DNC | 476 | 476 | DNC | N/A | African American |
| 37 | FTC | GSCAN | 1467 | 819 | 767 | DNC | 275/1192 | White European |
| 38 | FUSION | GSCAN | 1153 | 568 | 530 | DNC | N/A | White European |
| 39 | GECCO | GSCAN | 6459 | 2916 | 2876 | DNC | N/A | White European |
| 40 | GFG | GSCAN | 2994 | 1396 | 432 | DNC | N/A | White European |
| 41 | HRS | GSCAN | 6393 | 3303 | 3303 | DNC | N/A | White European |
| - | HRS – replication | GSCAN | DNC | 961 | 961 | DNC | N/A | African American |
| 42 | ID1000 | GSCAN | 803 | 366 | 373 | DNC | N/A | White European |
| 43 | MEC | GSCAN | 1903 | 1087 | 1082 | DNC | 396/1507 | White European |
| 44 | METSIM | GSCAN | 8146 | 1374 | 1370 | DNC | 8146/0 | White European |
| 45 | MHI | GSCAN | 6820 | 4391 | 4400 | DNC | 1950/4870 | White European |
| 46 | MN | GSCAN | DNC | 2043 | DNC | DNC | N/A | White European |
| 47 | NAGOZALC | GSCAN | 1038 | 671 | 646 | DNC | 187/851 | White European |
| 48 | NESCOG | GSCAN | 486 | 217 | 220 | DNC | N/A | White European |
| 49 | sardiNIA | GSCAN | 5069 | 1969 | 1967 | DNC | N/A | White European |
| 50 | TwinsUK | GSCAN | 878 | 358 | 358 | DNC | N/A | White European |
| 51 | UK Biobank (non-UK BiLEVE subset) | GSCAN | 73331 | 21525 | 21267 | 31748 (18084/13664) | 16538/56793 | White European |
| 52 | UK BiLEVE | GSCAN | 39480 | 19357 | 19357 | 19295(12836/6459) | 9945/29535 | White European |
| 53 | WHI | GSCAN | DNC | 6246 | 6236 | DNC | 2994 | White European |
| 54 | CCHS | CHDExome+ | 6287 (4021/2266) | DNC | DNC | 4010 (83/3927) | N/A | White European |
| 55 | CGPS | CHDExome+ | 11781 (7555/4226) | DNC | DNC | 7541 (4299/3242) | N/A | White European |
| 56 | CIHDS | CHDExome+ | 3434 (2074/1360) | DNC | DNC | DNC | N/A | White European |
| 57 | EPIC-CVD | CHDExome+ | 21475 (12477/8998) | 4680 (15.58/10.06) | 4548 (25.77/18.65) | 6015 (217/5798) | N/A | White European |
| 58 | INTERVAL | - | 36479 (15354/21125) | 14124 (9.85/7.72) | 12782 (8.59/10.25) | 15264 (12228/3036) | N/A | White European |
| 59 | PROSPER | CHDExome+ | 1279 (880/399) | DNC | DNC | 910 (588/322) | N/A | White European |
| 60 | PROMIS | CHDExome+ | 21831 (10008/11823) | 7913 (15.97/11.71) | 7623 (22.92/19.69) | 8509 (171/8338) | N/A | South Asian |
| 61 | BRAVE | CHDExome+ | 5543 (4252/1291) | 3144 (12.68/8.96) | 3090 (18.20/15.90) | 4022 (349/3673) | N/A | South Asian |
| 62 | MORGAM | CHDExome+ | DNC | 2684 (18.50/9.01) | DNC | DNC | N/A | White European |
| - | **Total** | - | 55 cohorts | 53 cohorts | 49 cohorts | 42 cohorts | - | - |

**Supp. Table 2:** Studies which contributed to primary analyses (at discovery stage), the consortia name, sample size of each dataset, and details of study specific genotyping platform and software used. CGSB: Consortium for the Genetics of Smoking Behaviour (Leicester); CHDExome+: Coronary Heart Disease Exome+ (Cambridge); GSCAN: GWAS & Sequencing Consortium of Alcohol and Nicotine (Colorado & Michigan). Affiliation with same consortia implies that a similar QC protocol and analysis plan was followed. DNC: Did not contribute; n: sample size; PC: principal component.

|  | **Cohort** | **Consortia** | **Genotyping Platform** | **Study-level software** | **Covariates used** | **Transformations** | **Analysis model** |
| --- | --- | --- | --- | --- | --- | --- | --- |
| 1 | Airwave | CGSB | HumanExome v1.1 | RareMetalWorker | Age, sex and top 4 PCs | Where available (see Supp. Table 1), quantitative traits (i.e. CPD and PY) were inverse normalised | CPD and PY were analysed using linear regression; and SI and SC were analysed using logistic regression |
| 2 | ASCOT – Scotland dataset | CGSB | Human OmniExpressExome v8.1 | RareMetalWorker | Age, sex and top 10 PCs |  |  |
| 3 | ASCOT – UK dataset | CGSB | HumanExome v1.1 | RareMetalWorker | Age, sex and top 10 PCs |  |  |
| 4 | 1958BC | CGSB | HumanExome v1.0 | RareMetalWorker | Age, sex and top 3 PCs |  |  |
| 5 | BRIGHT | CGSB | HumanExome v1.0 | RareMetalWorker | Age, sex and top 10 PCs |  |  |
| 6 | DIABNORD | CGSB | HumanExome v1.1 | RareMetalWorker | Age, sex and top 3 PCs |  |  |
| 7 | EFSOCH | CGSB | HumanExome v1.0 | RareMetalWorker | Age, sex and top 3 PCs |  |  |
| 8 | EGCUT – BMI dataset | CGSB | HumanExome v1.1 | RareMetalWorker | Age, sex and top 10 PCs |  |  |
| 9 | EGCUT – Controls dataset | CGSB | HumanExome v1.1 | RareMetalWorker | Age, sex and top 10 PCs |  |  |
| 10 | EGCUT - Height cases dataset | CGSB | HumanExome v1.1 | RareMetalWorker | Age, sex and top 10 PCs |  |  |
| 11 | EGCUT – Psoriasis cases dataset | CGSB | HumanExome v1.1 | RareMetalWorker | Age, sex and top 10 PCs |  |  |
| 12 | EGCUT - T2D cases dataset | CGSB | HumanExome v1.1 | RareMetalWorker | Age, sex and top 10 PCs |  |  |
| 13 | EGCUT – CoreExome dataset | CGSB | HumanCoreExome v1.1 | RareMetalWorker | Age, sex and top 10 PCs |  |  |
| 14 | EMBRACE | CGSB | Illumina ExomeChip v1.0 | RareMetalWorker | Age and top 3 PCs |  |  |
| 15 | Fenland | CGSB | HumanExome v1.0 | RareMetalWorker | Age, sex and top 10 PCs |  |  |
| 16 | FIA3 | CGSB | HumanExome v1.1 | RareMetalWorker | Age, sex and top 10 PCs |  |  |
| 17 | GS:SFHS | CGSB | HumanExome v1.0 | RareMetalWorker | Age, sex and top 3 PCs |  |  |
| 18 | GLACIER | CGSB | HumanExome v1.1 | RareMetalWorker | Age, sex and top 3 PCs |  |  |
| 19 | GoDARTS | CGSB | HumanExome v1.0 | RareMetalWorker | Age, sex and top 3 PCs |  |  |
| 20 | KORA F4 | CGSB | HumanExome v1.0 | RareMetalWorker | Age, sex and top 10 PCs |  |  |
| 21 | CROATIA-Korcula | CGSB | HumanExome v1.0 | RareMetalWorker | Age, sex and top 3 PCs |  |  |
| 22 | LBC1921 | CGSB | HumanExome v1.0 | RareMetalWorker | Age, sex and top 10 PCs |  |  |
| 23 | LBC1936 | CGSB | HumanExome v1.0 | RareMetalWorker | Age, sex and top 10 PCs |  |  |
| 24 | LifeLines | CGSB | HumanExome v1.1 | RareMetalWorker | Age, sex and top 5 PCs |  |  |
| 25 | LOLIPOP Exome chip dataset | CGSB | HumanExome v1.1 | RareMetalWorker | Age, sex and top 3 PCs |  |  |
| 26 | LOLIPOP OmniExpress chip dataset | CGSB | Human OmniExpressExome v8.1 | RareMetalWorker | Age, sex and top 3 PCs |  |  |
| 27 | LRGP | CGSB | HumanExome v1.1 | RareMetalWorker | Age, sex and top 3 PCs |  |  |
| 28 | OxBB | CGSB |  | RareMetalWorker | Age, sex and top 10 PCs |  |  |
| 29 | SEARCH – Breast Cancer dataset | CGSB | HumanExome v1.0 | RareMetalWorker | Age and top 3 PCs |  |  |
| 30 | SEARCH – Controls dataset | CGSB | HumanExome v1.0 | RareMetalWorker | Age, sex and top 3 PCs |  |  |
| 31 | SEARCH – Ovarian Cancer dataset | CGSB | HumanExome v1.0 | RareMetalWorker | Age and top 3 PCs |  |  |
| 32 | SHIP | CGSB | HumanExome v1.0 | RareMetalWorker | Age, sex and top 10 PCs |  |  |
| 33 | SIBS | CGSB | HumanExome v1.0 | RareMetalWorker | Age and top 3 PCs |  |  |
| 34 | UKHLS | CGSB | HumanCoreExome v1.0 | RareMetalWorker | Age, sex and top 10 PCs |  |  |
| 35 | ARIC | GSCAN | HumanExome v1.0 | RareMetalWorker | Age, sex and top 3 PCs | CPD was a categorical trait (1-4) with responses binned at 1-10 (1), 11-20 (2), 21-30 (3), and 31+ (4). The residuals for the quantitative traits were transformed using inverse normal transformation |  |
| 36 | COGA | GSCAN | HumanCoreExome v1.0 | RareMetalWorker | Age, sex and top 3 PCs |  |  |
| 37 | FTC | GSCAN | HumanCoreExome v1.0 | Rvtests | Age, age^2^, sex, BMI, assessment year and top 3 PCs |  |  |
| 38 | FUSION | GSCAN | HumanExome v1.0 | RareMetalWorker | Age, sex and top 3 PCs |  |  |
| 39 | GECCO | GSCAN | HumanExome v1.0 | RareMetalWorker | Age, sex and top 3 PCs |  |  |
| 40 | GFG | GSCAN | Illumina HumanCoreExome array with custom content | Rvtests | Age, age^2^, sex and top 3 PCs |  |  |
| 41 | HRS | GSCAN | HumanExome v1.0 | RareMetalWorker | Age, sex and top 3 PCs |  |  |
| - | HRS - replication | GSCAN | HumanExome v1.0 | RareMetalWorker | Age, sex and top 10 PCs |  |  |
| 42 | ID1000 | GSCAN | HumanExome v1.0 | RareMetalWorker | Age, sex and top 3 PCs |  |  |
| 43 | MEC | GSCAN | HumanExome v1.0 | RareMetalWorker | Age, sex and top 10 PCs |  |  |
| 44 | METSIM | GSCAN | HumanExome v1.0 | RareMetalWorker | Age, sex and top 3 PCs |  |  |
| 45 | MHI | GSCAN | HumanExome v1.0 | Rvtests | Age, age^2^, sex, enrolment date and top 10 PCs |  |  |
| 46 | MN | GSCAN | HumanExome v1.0 | RareMetalWorker | Age, sex and top 3 PCs |  |  |
| 47 | NAGOZALC | GSCAN | HumanCNV370-quad V3 | RareMetalWorker | Age, sex and top 3 PCs |  |  |
| 48 | NESCOG | GSCAN | HumanExome v1.0 | RareMetalWorker | Age, sex and top 3 PCs |  |  |
| 49 | sardiNIA | GSCAN | HumanExome v1.0 | RareMetalWorker | Age, sex and top 3 PCs |  |  |
| 50 | TwinsUK | GSCAN | HumanExome v1.0 | RareMetalWorker | Age, sex and top 3 PCs |  |  |
| 51 | UK Biobank (non-UK BiLEVE subset) | GSCAN | UK Biobank Axiom Array | Rvtests | Age, age^2^, sex and top 10 PCs |  |  |
| 52 | UK BiLEVE | GSCAN | UK BiLEVE Axiom Array | Rvtests | Age, age^2^, sex and top 10 PCs |  |  |
| 53 | WHI | GSCAN | HumanExome v1.0 | RareMetalWorker | Age, sex and top 3 PCs |  |  |
| 54 | CCHS | CHDExome+ | HumanExome v1.1 | RareMetalWorker | Age, sex and top 3 PCs | Where available (see Supp. Table 1), quantitative traits were inverse normalised | All traits were analysed using linear mixed models |
| 55 | CGPS | CHDExome+ | HumanExome v1.1 | RareMetalWorker | Age, sex and top 3 PCs |  |  |
| 56 | CIHDS | CHDExome+ | HumanExome v1.1 | RareMetalWorker | Age, sex and top 3 PCs |  |  |
| 57 | EPIC-CVD | CHDExome+ | HumanExome v1.1 | RareMetalWorker | Age, sex and top 3 PCs |  |  |
| 58 | INTERVAL | - | UK Biobank Axiom Array | RareMetalWorker | Age, age^2^, sex, blood donation centre, BMI and top 3 PCs |  |  |
| 59 | PROSPER | CHDExome+ | HumanExome v1.1 | RareMetalWorker | Age, sex and top 3 PCs |  |  |
| 60 | PROMIS (South Asian samples) | CHDExome+ | HumanExome v1.1 | RareMetalWorker | Age, sex and top 3 PCs |  |  |
| 61 | BRAVE (South Asian samples) | CHDExome+ | HumanExome v1.1 | RareMetalWorker | Age, sex and top 3 PCs |  |  |
| 62 | MORGAM | CHDExome+ | HumanExome v1.1 | RareMetalWorker | Age, sex and top 3 PCs |  |  |
| - | **Meta-analysis** | - | - | RAREMETAL |  |  |  |

**Supp. Table 3:** Association of the 14 SNPs previously identified smoking behaviour loci in the discovery stage cohorts. For each variant, the result is presented for the smoking behaviour related trait for which it was first reported. SNPs with *P*<5x10^-8^ are in **bold**. r^2^: r^2^ value between the Exome chip proxy SNP and the previously reported SNP in White European samples of the 1000 Genomes project; non-Ex: Non- Exome chip SNP; NA: A proxy SNP could not be found for the previously reported SNP (r^2^≥0.3); SI: Smoking initiations; SC: Smoking cessation; CPD: Cigarettes per day; PY: Pack-years.

| **Reported SNP ID**  **(effect/alternative allele)** | **Chr:Pos (hg19)** | **Exome-chip proxy or UK Biobank Axiom Array SNP ID** | **Proxy Chr:Pos** | **Proxy SNP effect/ alternative allele (consequence)** | **Gene** | **Trait** | **Discovery P-value of proxy SNP** | **Replication stage P-value (beta/se)** | **Combined meta-analysis P-value of proxy SNP** | **r^2^** | **References** |
| --- | --- | --- | --- | --- | --- | --- | --- | --- | --- | --- | --- |
| rs1051730 (A/G) | 15:78894339 | rs1051730 | 15:78894339 | A/G (synonymous) | 15q25 (*CHRNA3*) | CPD | **2.17x10^-32^** | **2.6x10^-81^** (0.101/ 0.0052) | **5.5x10^-121^** | Same SNP | The Tobacco and Genetics Consortium, 2010^6^ |
|  |  |  |  |  |  | PY | **2.83E^-21^** | NA | NA | - | - |
| rs215605 (G/T) | 7:32336965 | rs215607 | 7:32338337 | A/G (missense) | 7p14 (*PDE1C*) | CPD | 0.017 | 0.024  (-0.013/ 0.0059) | 9.0x10^-4^ | 0.46 | Thorgeirsson *et al*, 2010^7^ |
|  |  |  |  |  |  | PY | 5.5x10^-8^ | NA | NA | - | - |
| rs13280604 (G/A) | 8:42559586 | rs6474412 | 8:42550498 | T/C (intergenic) | 8p11 (*CHRNB3*) | CPD | **1.3x10^-11^** | **9.8x10^-13^** (0.043/ 0.0060) | **2.2x10^-21^** | 1 | Thorgeirsson *et al*, 2010^7^ |
|  |  |  |  |  |  | PY | 1.25x10^-5^ | NA | NA | - | - |
| rs1329650 (G/T) | 10:93348120 | rs1329650 | 10:93348120 | G/T (intergenic) | *LOC100188947* | CPD | 0.068 | 0.51 (0.0037/ 0.0056) | 0.081 | Same SNP | The Tobacco and Genetics Consortium, 2010^6^ & Thakur *et al*, 2012^8^ |
|  |  |  |  |  |  | PY | 0.063 | NA | NA | - | - |
| rs3733829 (G/A) | 19:41310571 | rs3733829 | 19:41310571 | G/A (intronic) | *EGLN2* | CPD | 0.00022 | 1.1x10^-6^ (0.025/ 0.0052) | **1.66x10^-9^** | Same SNP | The Tobacco and Genetics Consortium, 2010^6^ & Bloom *et al*, 2014^9^ |
|  |  |  |  |  |  | PY | 0.016 | NA | NA | - | - |
| rs7937 (C/T) | 19:41302706 | rs7937 | 19:41302706 | C/T (intronic) | 19q13 (*RAB4B*) | CPD | **5.35x10^-11^** | **8.7x10^-14^**  (-0.037/ 0.0050) | NA | Same SNP | Thorgeirsson *et al*, 2010^7^ & Timofeeva *et al*, 2011^10^ |
|  |  |  |  |  |  | PY | **8.18x10^-9^** | NA | NA | - | - |
| rs3025343 (A/G) | 9:136478355 | rs3025343 | 9:136478355 | A/G (intergenic) | *DBH* | SC | 0.00028 | **3.2x10^-10^** (0.039/ 0.0062) | **3.94x10^-12^** | Same SNP | The Tobacco and Genetics Consortium, 2010^6^ & Siedlinski *et al*, 2011^11^ |
| rs6265 (T/C) | 11:27679916 | rs6265 | 11:27679916 | T/C (missense) | *BDNF* | SI | 8.59x10^-6^ | **2.9x10^-8^**  (-0.019/ 0.0034) | **8.43x10^-12^** | Same SNP | The Tobacco and Genetics Consortium, 2010^6^ |
| rs4466874 (C/T) | 11:112861434 | rs4144892  (non-Ex) | 11:112866456 | T/C (intronic) | *NCAM1* | SI | **4.7x10^-10^** | **6.1x10^-17^** (0.023/ 0.0027) | **7.26x10^-25^** | 1 | Wain *et al*, 2015^12^ |
| rs10193706 (C/A) | 2:146316319 | rs10427255 | 2:146125523 | T/C (intergenic) | *TEX41/PABPC1P2* | SI | **3.06x10^-14^** | **6.2x10^-10^**  (-0.0166/ 0.0027) | **2.97x10^-22^** | 0.40 | Wain *et al*, 2015^12^ |
| rs61784651 (T/C) | 1:99445471 | rs61784651  (non-Ex) | 1:99445471 | T/C (intergenic) | *LPPR5* | SI | 0.00010 | 3.3x10^-3^ (0.0105/ 0.0036) | 0.0071 | Same SNP | Wain *et al*, 2015^12^ |
| rs10807199 (T/C) | 6:38901867 | rs9296270 (non-Ex) | 6:38903095 | A/G (intronic) | *DNAH8* | SI | 0.0012 | 0.74 (0.0009/ 0.0027) | 0.0109 | 1 | Wain *et al*, 2015^12^ |
| rs143125561 (C/CACGG) | 20:31162590-31162591 | rs4911241 | 20:31140165 | T/C (intronic) | *NOL4L* | SI | 7.22x10^-5^ | 6.4x10^-8^ (0.0170/ 0.0031) | **2.94x10^-10^** | 0.91 | Wain *et al*, 2015^12^ |
| rs2273500 (C/T) | 20:61986949 | rs2273506 | 20:61990939 | A/G (synonymous) | *CHRNA4* | Fagerström test (CPD) | 5.41x10^-5^ | 0.003 (0.030/ 0.0101) | 8.92x10^-7^ | 0.40 | Hancock *et al*, 2015^13^ (& Wain *et al*, 2015^12^) |

**Supp. Table 4:** Results from sensitivity analyses, and consortium-specific association studies for each novel SNP (discovery stage). CHDExome+ consortium did not contribute to X chromosome analyses. Same: Same *P*-value as in Primary analysis. MAC, effect size (β) and 95% confidence interval (CI) of rs141611945 (*ATF6*) added for additional information on this rare SNP – to add evidence as (internal) replication. * The rare nonsynonymous *ATF6* SNV, rs141611945, associated with CPD in the discovery stage of this study, was only polymorphic in six studies, with a total MAC=9 across all 129,000 individuals. The variant was not available in UK Biobank. rs141611945 is more common in African ancestries (1.2%), but we were unable to ascertain sufficient numbers of African-ancestry individuals (n=1,437) to replicate the association.

| **Trait** | **Gene** | **SNP ID** | **Chr:Pos** | ***P*-value in Primary analysis** | ***P*-value excluding all UK Biobank samples** | ***P*-value excluding all UK Biobank and South Asian samples** | ***P*-value in CGSB** | ***P*-value in GSCAN** | ***P*-value in CHDExome+ plus INTERVAL samples** | ***P*-value in South Asian samples only** | ***P*-value excl. UK BiLEVE samples** |
| --- | --- | --- | --- | --- | --- | --- | --- | --- | --- | --- | --- |
| CPD | *ATF6** | rs141611945 | 1:161771868 | 2.95x10^-7^ (n=128,746; β=1.71; 95% CI: 2.36-1.05) | Same | Same | 0.00017 (n=26,506, MAC=6; β=1.53; 95% CI: 2.33-0.73) | 0.0053 (n=69,695, MAC=2; β=1.97; 95% CI: 3.36-0.59) | 0.025 (n=32,545, MAC=1; β=2.24; 95% CI: 4.24-0.28) | NA | NA |
| CPD | *GPR101* | rs1190736 | X:136113464 | **1.40x10^-11^** (n=99,037) | 3.28x10^-7^ (n=90,398) | Same as left | 0.0010 (n=26,499) | **3.42x10^-9^** (n=51,050) | NA | NA | NA |
| SI | *REV3L* | rs462779 | 6:111695887 | **4.52x10^-8^** (n=346,682) | 1.62x10^-6^ (n=233,871) | 3.14x10^-7^ (n=212,040) | 1.20x10^-5^ (n=78,048) | 0.0013 (n=165,368) | 0.0247 (n=103,266) | 0.754 (n=21,831) | NA |
| SI | *SMG6* | rs216195 | 17:2203167 | **2.80x10^-8^** (n=335,406) | 3.34x10^-7^ (n=222,595) | 8.22x10^-8^ (n=200,937) | 0.0013 (n=78,056) | 2.04x10^-5^ (n=154,822) | 0.00245 (n=102,528) | 0.542 (n=21,658) | NA |
| SI | *PJA1* | rs11539157 | X:68381264 | **1.39x10^-11^** (n=289,917) | **4.53x10^-9^** (n=230,072) | Same as left | 8.73x10^-7^ (n=78,040) | 3.09x10^-7^ (n=108,512) | NA | NA | NA |
| **Non-Exome chip SNVs** | | | | | | | | | | | |
| SI | *TMEM182* | rs12616219 | 2:104352495 | 5.49x10^-8^ (n=112,811) | NA | NA | NA | Same | NA | NA | 0.00027 |
| SI | *ZSCAN9* | rs462779 | 6:28168033 | **4.95x10^-8^** (n=112,811) | NA | NA | NA | Same | NA | NA | 0.00051 |
| SI | *GAPVD1* | rs2841334 | 9:128122320 | **2.28x10^-8^** (n=112,811) | NA | NA | NA | Same | NA | NA | 5.26x10^-5^ |
| SC | *TOB2* | rs202664 | 22:41813886 | **1.02x10^-8^** (n=51,043) | NA | NA | NA | Same | NA | NA | 2.89x10^-7^ |
| SI | *BCL11A* | rs11895381 | 2:60053727 | **5.62x10^-9^** (n=112,811) | NA | NA | NA | Same | NA | NA | 4.44x10^-6^ |
| SI | *CNNM2* | rs12780116 | 10:104821946 | **9.19x10^-10^** (n=112,811) | NA | NA | NA | Same | NA | NA | 9.61x10^-5^ |

**Supp. Table 5:** Single variant association results for all novel and previously reported SNVs across all four traits (discovery stage). SNVs which reach *P*<5x10^-8^ are highlighted in **bold**. NA: Reported SNP (or a proxy) not available in our study. Direction of effect provided in parentheses for all variants reaching *P*<0.05.

| **Reported SNP ID**  **(effect/alternative allele)** | **Chr:Pos (hg19)** | **Gene** | ***P*-value for SI** (direction of effect) | ***P*-value for CPD** (direction of effect) | ***P*-value for PY** (direction of effect) | ***P*-value for SC** (direction of effect) | **Notes** |
| --- | --- | --- | --- | --- | --- | --- | --- |
| **Novel SNVs identified in this study** | | | | | | | |
| rs141611945 (G/A) | 1:161771868 | *ATF6* | 0.58 | 2.95x10^-7^ (+) | 0.00015 (+) | 0.866 | - |
| rs1190736 (A/C) | X:136113464 | *GPR101* | 0.13 | **1.40x10^-11^** (-) | **4.98x10^-9^** (-) | 0.503 | - |
| rs462779 (A/G) | 6:111695887 | *REV3L* | **4.52x10^-8^** (-) | 0.651 | 0.545 | 0.042 (+) | - |
| rs216195 (G/T) | 17:2203167 | *SMG6* | **2.80x10^-8^** (-) | 0.378 | 0.628 | 0.446 | - |
| rs11539157 (A/C) | X:68381264 | *PJA1* | **1.40x10^-11^** (+) | 0.087 | 0.0017 (+) | 0.034 (-) | - |
| rs12616219 (A/C) | 2:104352495 | *TMEM182* | 5.49x10^-8^ (-) | 0.495 | 0.814 | 0.201 | - |
| rs1150691 (G/A) | 6:28168033 | *ZSCAN9* | **4.95x10^-8^** (-) | 0.523 | 0.499 | 0.415 | - |
| rs2841334 (A/G) | 9:128122320 | *GAPVD1* | **2.28x10^-8^** (-) | 0.088 | 0.260 | 0.0081 (-) | - |
| rs202664 (C/T) | 22:41813886 | *TOB2* | 0.26 | 0.865 | 0.416 | **1.02x10^-8^** (-) | - |
| rs11895381 (A/G) | 2:60053727 | *BCL11A* | **5.62x10^-9^** (-) | 0.467 | 0.268 | 0.491 | - |
| rs12780116 (A/G) | 10:104821946 | *CNNM2* | **9.19x10^-10^** (+) | 0.305 | 0.635 | 0.884 | - |
| **Previously reported SNVs** | | | | | | | |
| rs1051730 (A/G) | 15:78894339 | 15q25 (*CHRNA3*) | 0.23 | **2.17x10^-32^** (+) | **2.83x10^-21^** (+) | 0.043 (+) | - |
| rs215605 (G/T) | 7:32336965 | 7p14 (*PDE1C*) | 0.014 (+) | 0.0099 (+) | **5.41x10^-6^** (+) | 0.033 (+) | Results for rs215607 provided in **Supp. Table 3** |
| rs13280604 (G/A) | 8:42559586 | 8p11 (*CHRNB3*) | 0.49 | 0.0012 (-) | 0.064 | 0.97 | - |
| rs1329650 (T/G) | 10:93348120 | *LOC100188947* | 0.010 (-) | 0.068 | 0.063 | 0.40 | - |
| rs3733829 (G/A) | 19:41310571 | *EGLN2* | 0.48 | 0.00022 (+) | 0.016 (+) | 0.936 | - |
| rs7937 (T/C) | 19:41302706 | 19q13 (*RAB4B*) | 0.75 | **5.35x10^-11^** (+) | **8.18x10^-9^** (+) | 0.0054 (-) | - |
| rs3025343 (A/G) | 9:136478355 | *DBH* | 0.010 (+) | **2.93x10^-9^** (+) | **1.29x10^-14^** (+) | 0.00028 (-) | - |
| rs6265 (T/C) | 11:27679916 | *BDNF* | 8.59x10^-6^ (-) | 0.028 (-) | 0.0087 (-) | 0.228 | - |
| rs4466874 (C/T) | 11:112861434 | *NCAM1* | **4.73x10^-10^** (+) | 0.675 | 0.398 | 0.108 | Results are for rs4144892 (r^2^= 1; T/C) |
| rs10193706 (C/A) | 2:146316319 | *TEX41/PABPC1P2* | **3.07x10^-14^** (-) | 0.955 | 0.176 | 0.522 | Results are for rs10427255 (r^2^= 0.49; T/C) |
| rs61784651 (T/C) | 1:99445471 | *LPPR5* | 0.0001 (+) | 0.121 | 0.580 | 0.689 | - |
| rs10807199 (T/C) | 6:38901867 | *DNAH8* | 0.00125 (+) | 0.896 | 0.612 | 0.754 | Results are for rs9296270 (r^2^= 1; A/G) |
| rs143125561 (C/CACGG) | 20:31162590-31162591 | *NOL4L* | NA | NA | NA | NA | - |
| rs2273500 (C/T) | 20:61986949 | *CHRNA4* | 0.749 | 5.41x10^-5^ (+) | 0.00092 (+) | 0.511 | Results are for rs2273506 (r^2^= 0.32; A/G) |

**Supp. Table 6:** Results for the top four genes from gene-based analyses. *P*-values obtained from each of the collapsing methods utilised, and the variants which were collapsed to produce the overall ‘Gene *P*-value’ are provided. RsIDs of variants with a MAF>0.01 were included. PY: Pack-years; WST: Weighted sum test; DoE: Direction of effect from the burden test. Conditional analyses were performed to ascertain if the associations below were attributable to more than one SNV. The SNV used to condition on (which is the SNV with the smallest *P*-value in the gene) is listed in ‘SNV to condition on’.

| **Trait** | **Gene** | **SNVs for gene tests** | **MAF** | **SNV *P*** | **Gene *based test P*-value** | | **Conditional gene-based tests MAF=0.05** | |
| --- | --- | --- | --- | --- | --- | --- | --- | --- |
|  |  |  |  |  | **MAF<0.05** [DoE] | **MAF<0.01** | **SNV to condition on** | ***P*-values MAF<0.05 (MAF<0.01)** |
| CPD | *CRCP* | 7:65617235:T:C | 4.00E-04 | 0.0413 | Burden: 7.24x10^-4^  WST: 1.94x10^-4^  SKAT: 0.0177  [-] | Burden: 7.24x10^-4^  WST: 1.94x10^-4^  SKAT: 0.0177  [-] | 7:65617261 | Burden: 9.37x10^-3^ (9.37x10^-3^)  WST: 4.31x10^-3^ (4.31x10^-3^)  SKAT: 0.0333 (0.0333) |
|  |  | 7:65617261:A:G | 1.00E-04 | 0.0128 |  |  |  |  |
|  |  | 7:65617327:G:A | 8.00E-05 | 0.0406 |  |  |  |  |
| CPD | *CHRNA5* | 15:78873272:T:G | 2.76E-04 | 0.3075 | Burden: **3.38x10^-8^**  WST: 1.57x10^-4^  SKAT: **2.56x10^-8^**  [+] | Burden: 0.0741  WST: 0.0479  SKAT: 0.416 | rs2229961 | Burden: 0.28 (0.084)  WST: 0.0521 (0.05)  SKAT: 0.75 (0.51) |
|  |  | 15:78880752:G:A (rs2229961) | 0.0167 | 2.67E-08 |  |  |  |  |
|  |  | 15:78882233:A:G | 3.46E-05 | 0.7181 |  |  |  |  |
|  |  | 15:78882331:A:G | 1.45E-04 | 0.7805 |  |  |  |  |
|  |  | 15:78882446:C:T | 1.60E-04 | 0.5017 |  |  |  |  |
|  |  | 15:78882682:C:G | 4.43E-05 | 0.3140 |  |  |  |  |
|  |  | 15:78882694:A:G | 1.65E-04 | 0.2746 |  |  |  |  |
|  |  | 15:78882726:C:T | 2.01E-04 | 0.1565 |  |  |  |  |
|  |  | 15:78882797:T:C | 3.94E-04 | 0.3795 |  |  |  |  |
|  |  | 15:78882821:T:A | 1.86E-04 | 0.1655 |  |  |  |  |
|  |  | 15:78882920:C:T | 2.29E-05 | 0.9233 |  |  |  |  |
|  |  | 15:78882934:C:T | 8.25E-06 | 0.3324 |  |  |  |  |
|  |  | 15:78885574:T:A (rs76071148) | 0.0176 | 0.7520 |  |  |  |  |
| PY | *MMP17* | 12:132322801:C:A | 2.22E-05 | 0.7454 | Burden: 2.28x10^-5^  WST: 8.50x10^-4^  SKAT: 6.44x10^-4^  [-] | Burden: 4.96x10^-3^  WST: 0.0103  SKAT: 0.0725 | rs4964883 | Burden: 4.45x10^-3^ (4.45x10^-3^)  WST: 9.81x10^-3^ (9.82x10^-3^)  SKAT: 0.0655 (0.0655) |
|  |  | 12:132322812:C:A | 4.74E-03 | 0.0828 |  |  |  |  |
|  |  | 12:132323249:G:A | 1.41E-05 | 0.6916 |  |  |  |  |
|  |  | 12:132323250:C:G (rs4964883) | 0.0178 | 0.0017 |  |  |  |  |
|  |  | 12:132325122:C:T | 1.57E-04 | 0.0116 |  |  |  |  |
|  |  | 12:132325135:G:A | 2.01E-03 | 0.4579 |  |  |  |  |
|  |  | 12:132325155:G:A | 4.95E-05 | 0.1567 |  |  |  |  |
|  |  | 12:132325204:T:G | 2.13E-04 | 0.2598 |  |  |  |  |
|  |  | 12:132326297:C:T | 1.68E-04 | 0.1078 |  |  |  |  |
|  |  | 12:132328566:C:T | 2.95E-04 | 0.2026 |  |  |  |  |
|  |  | 12:132334379:G:A | 3.19E-04 | 0.7728 |  |  |  |  |
|  |  | 12:132334403:G:A | 8.98E-05 | 0.4139 |  |  |  |  |
|  |  | 12:132334430:G:A | 5.99E-04 | 0.9678 |  |  |  |  |
|  |  | 12:132334460:A:G | 3.68E-03 | 0.0332 |  |  |  |  |
|  |  | 12:132335602:T:C | 8.47E-05 | 0.9274 |  |  |  |  |
|  |  | 12:132335664:C:T | 9.59E-04 | 0.9049 |  |  |  |  |
|  |  | 12:132335685:G:A | 5.02E-03 | 0.4770 |  |  |  |  |
| PY | *CHRNA2* | 8:27320526:G:T | 4.96E-03 | 0.0399 | Burden: 6.40x10^-4^  WST: 0.19  SKAT: 0.0026  [+] | Burden: 0.043  WST: 0.75  SKAT: 0.041 | rs56229264 | Burden: 0.04 (0.04)  WST: 0.73 (0.73)  SKAT: 0.038 (0.038) |
|  |  | 8:27320528:C:T | 8.42E-05 | 0.5917 |  |  |  |  |
|  |  | 8:27320726:C:T | 4.31E-04 | 0.6571 |  |  |  |  |
|  |  | 8:27321189:G:A (rs56229264) | 0.01606 | 0.0063 |  |  |  |  |
|  |  | 8:27324812:C:T | 6.47E-05 | 0.5914 |  |  |  |  |
|  |  | 8:27327391:G:A | 7.86E-06 | 0.0293 |  |  |  |  |
|  |  | 8:27327432:G:A | 5.94E-04 | 0.1764 |  |  |  |  |

**Supp. Table 7: Results from Mendelian Randomization (MR) analyses to assess causal effects of smoking on BMI, schizophrenia, and education attainment.** Three complementary approaches were performed including i) MR-Egger, ii) weighted median iii) inverse variance weighted regression. The analyses were performed using the R package Mr Base using MR-Base ID: 2 for BMI, MR-Base ID: 22 for schizophrenia and MR-Base ID: 1001 for educational attainment. We also performed sensitivity analyses to check for reverse causality.

A. Smoking Initiation (SI) with BMI, schizophrenia, and education attainment using smoking initiation associated SNVs as instrumental variables (IVs). The *P*-value for the intercept for MR-Egger is provided in parentheses.

| **MR Method** | **Number of IVs** | **Beta (SE)** | ***P*-VALUE** |
| --- | --- | --- | --- |
| **SI → BMI** | | | |
| MR Egger | 43 | -0.31 (0.12) | 0.013 (0.001) |
| Weighted median | 43 | -0.043 (0.033) | 0.19 |
| Inverse variance weighted | 43 | 0.061 (0.065) | 0.35 |
| **SI → Schizophrenia** | | | |
| MR Egger | 46 | 0.199 (0.32) | 0.54 (0.57) |
| Weighted median | 46 | 0.083 (0.099) | 0.403 |
| Inverse variance weighted | 46 | 0.36 (0.15) | 0.014 |
| **SI -> Education Attainment** | | | |
| MR Egger | 47 | -0.075 (0.06) | 0.202(0.39) |
| Weighted median | 47 | -0.087 (0.02) | 3.20e-5 |
| Inverse variance weighted | 47 | -0.120 (0.03) | 1.62e-6 |

B. Assessment of potential reverse causation on Smoking Initiation (SI) induced by BMI, schizophrenia, and education attainment using BMI, schizophrenia, and education attainment associated SNVs as instrumental variables (IVs)

| **MR Method** | **Number of IVs** | **Beta (SE)** | ***P*-VALUE** |
| --- | --- | --- | --- |
| **BMI→ SI** | | | |
| MR Egger | 60 | 0.022 (0.023) | 0.34 (0.81) |
| Weighted median | 60 | 0.024 (0.018) | 0.17 |
| Inverse variance weighted | 60 | 0.018 (0.015) | 0.23 |
| **Schizophrenia → SI** | | | |
| MR Egger | 8 | 0.196 (0.13) | 0.19 (0.13) |
| Weighted median | 8 | 0.00038 (0.025) | 0.99 |
| Inverse variance weighted | 8 | -0.027 (0.028) | 0.33 |
| **Education Attainment → SI** | | | |
| MR Egger | 10 | -0.81 (0.76) | 0.32 (0.99) |
| Weighted median | 10 | -0.13 (0.09) | 0.16 |
| Inverse variance weighted | 10 | -0.27 (0.16) | 0.088 |

C. Assessment of potential causal effect of Cigarettes per day (CPD) on body mass index (BMI), schizophrenia, and education attainment using cigarettes per day associated SNPs as instrumental variables

| **MR Method** | **Number of IVs** | **Beta(SE)** | ***P*-VALUE** |
| --- | --- | --- | --- |
| **CPD → BMI** | | | |
| MR Egger | 9 | -0.18 (0.062) | 0.021 (0.033) |
| Weighted median | 9 | -0.087 (0.033) | 0.0088 |
| Inverse variance weighted | 9 | -0.051 (0.048) | 0.29 |
| **CPD → Schizophrenia** | | | |
| MR Egger | 12 | 0.49 (0.29) | 0.12 (0.044) |
| Weighted median | 12 | 0.44 (0.13) | 0.00095 |
| Inverse variance weighted | 12 | 0.31 (0.17) | 0.068 |
| **CPD → Education Attainment** | | | |
| MR Egger | 11 | 0.035 (0.044) | 0.45 (0.041) |
| Weighted median | 11 | -0.041 (0.022) | 0.066 |
| Inverse variance weighted | 11 | -0.049 (0.031) | 0.11 |

D. Assessment of potential reverse causation on CPD induced by BMI, schizophrenia, and education attainment using BMI, schizophrenia, and education attainment associated SNVs as instrumental variables (IVs)

| **MR Method** | **Number of IVs** | **Beta (SE)** | ***P*-VALUE** |
| --- | --- | --- | --- |
| **BMI → CPD** | | | |
| MR Egger | 60 | 0.015 (0.047) | 0.74 (0.47) |
| Weighted median | 60 | 0.061 (0.041) | 0.14 |
| Inverse variance weighted | 60 | 0.043 (0.028) | 0.13 |
| **Schizophrenia → CPD** | | | |
| MR Egger | 8 | 0.303 (0.72) | 0.69 (0.96) |
| Weighted median | 8 | -0.0099 (0.05) | 0.85 |
| Inverse variance weighted | 8 | 0.26 (0.16) | 0.11 |
| **Education Attainment → CPD** | | | |
| MR Egger | 8 | -1.12 (0.77) | 0.19 (0.28) |
| Weighted median | 8 | -0.079 (0.28) | 0.78 |
| Inverse variance weighted | 8 | -0.246 (0.209) | 0.24 |

**Supp. Table 8:** Evaluation of potential collider bias in UK Biobank (UKBB) analyses. We performed two sensitivity analyses to understand whether collider bias influenced our results: i) performing meta-analysis without UK BiLEVE, the component of the UK Biobank that is enriched heavy smokers, ii) performing UK Biobank analysis without adjusting for genotyping array. We compared these results with our meta-analysis which adjusted for UK BiLEVE and UK Biobank Axiom arrays. The magnitude of the genetic effect estimates are very comparable for the three analyses, including the results with and without the UK BiLEVE samples. We used CPD as the outcome.

| **rsID (Exome-chip ID)** | **Chr:Position (REF/ALT)** | **Meta-analysis including UK BiLEVE** | | **Meta-analysis without UK BiLEVE** | | **UKBB without adjustment for array** |
| --- | --- | --- | --- | --- | --- | --- |
|  |  | **Beta (SE)** | ***P*-VALUE** | **Beta (SE)** | ***P*-VALUE** | **Beta (SE)** |
| rs141611945 (exm118559) | 1:161771868 (A/G) | 1.7 (0.33) | 2.95×10^-7^ | 1.7 (0.33) | 6.1×10^-7^ | 1.2 (0.46) |
| rs1190736 (exm1659559) | X:136113464 (C/A) | -0.028 (0.0055) | 3.45×10^-7^ | -0.016 (0.0045) | 3.2x10^-4^ | -0.019 (0.0034) |
| rs2960306 (exm383568) | 4:2990499 (G/T) | -0.017 (0.0041) | 4.33×10^-5^ | -0.012 (0.0045) | 5.3x10^-3^ | -0.017 (0.0044) |
| rs8102683 | 19:41363765 | 0.062 (0.0076) | 4.5×10^-16^ | 0.055 (0.010) | 8.6×10^-8^ | 0.044 (0.0031) |
| rs28399442 | 19:41354458 (C/A) | -0.18 (0.025) | 2.3×10^-12^ | -0.18 (0.035) | 2.7×10^-7^ | -0.17 (0.014) |
| rs3865453 | 19:41338556 (C/T) | -0.078 (0.014) | 3.0×10^-8^ | -0.074 (0.019) | 1.1×10^-4^ | -0.068 (0.0083) |
| rs938682 | 15:78882925 (G/A) | 0.094 (0.0043) | 8.8×10^-108^ | 0.099 (0.0046) | 1.6×10^-100^ | 0.085 (0.0044) |

## Individual study descriptions

This section describes study-specific characteristics. All participants provided written informed consent and studies were approved by local Research Ethics Committees and/or Institutional Review boards.

**Airwave (Airwave Health Monitoring Study)** is a large-scale cohort of police employees. Study details are given elsewhere^14^.

**ASCOT (Anglo Scandinavian Cardiac Outcomes Trial)** is a prospective, randomized, open, blinded endpoint trial for which details are given elsewhere^15^.

Details of the **1958BC** (**British 1958 Birth Cohort)** study have been previously reported^16^.

**BRIGHT (The British Genetics of Hypertension)** study is a hypertension case-control study. Study details are given elsewhere^17^.

The CROATIA study was initiated to investigate the use of isolated rather than urban populations for the identification of genes associated with medically-relevant quantitative traits. Three cohorts have been recruited as part of the CROATIA study, of which one, **CROATIA-Korcula^18^** has been used in these analyses. CROATIA-Korcula was recruited from 2007 to 2008 from the town of Korcula and the villages of Lumbarda, Zrnovo and Racisce on the island of Korcula, Croatia with 969 adults aged 18-98 agreeing to participate. Participants donated blood for DNA extraction and biochemical measurements as well as undergoing some anthropometric measurements and physiological tests to measure traits such as height, weight and blood pressure, and finally completing several questionnaires relating to general health, medical history, diet and lifestyle. Ethical approval was obtained from appropriate regulatory bodies in both Scotland and Croatia and participants gave informed consent prior to joining the study.

The **DIABNORD**, **FIA3 (FörstagångsInsjuknande i hjärtinfarkt i AC-län 3; English: First myocardial Infarction in AC county 3)** and **GLACIER (The Gene-Lifestyle interactions And Complex traits Involved in Elevated disease Risk)** studies are nested within the Västerbotten Health Survey, which are part of the Northern Sweden Health and Disease Study, a population-based prospective cohort study from northern Sweden. Study details are given elsewhere^19^.

**EFSOCH (The Exeter Family Study of Childhood Health)** is a prospective study of parents and children from a consecutive birth cohort. Study details are given elsewhere^20^.

**EGCUT (Estonian Genome Project of University of Tartu)** is a population-based biobank of the Estonian Genome Project of University of Tartu. The project is conducted according to the Estonian Gene Research Act and all participants have signed the broad informed consent (www.biobank.ee). In total, 52,000 individuals aged 18 years or older participated in this cohort (33% men, 67% women). The population distributions of the cohort reflect those of the Estonian population (83% Estonians, 14% Russians and 3% other). General practitioners (GP) and physicians in the hospitals randomly recruited the participants and a PC assisted interview was conducted for 1–2 hours. Data on demographics, genealogy, educational and occupational history, lifestyle and anthropometric and physiological data were assessed. Study details are given elsewhere (as Estonian Biobank)^21^.

**EMBRACE (Epidemiological Study of Familial Breast Cancer)** aims to “obtain prospective estimates of cancer incidence in BRCA1/2 mutation carriers; determine lifestyle factors which may modify cancer risk; study modifying genes; examine efficacy of interventions (mastectomy, oophorectomy etc) and provide a basis for future intervention trials”. Study details can be found at [ccge.medschl.cam.ac.uk/embrace](http://ccge.medschl.cam.ac.uk/embrace/).

**Fenland (Fenland Study)** is a population-based cohort study designed to investigate the association between genetic and lifestyle environmental factors and the risk of obesity, insulin sensitivity, hyperglycemia and related metabolic traits in men and women aged 30 to 55 yrs. Volunteers were recruited from General Practice sampling frames in the Fenland, Ely and Cambridge areas of the Cambridgeshire Primary Care Trust in the U.K.

The **Generation Scotland: Scottish Family Health Study** (**GS:SFHS**) is a collaboration between the Scottish Universities and the NHS, funded by the Chief Scientist Office of the Scottish Government. GS:SFHS is a family-based genetic epidemiology cohort with DNA, other biological samples (serum, urine and cryopreserved whole blood) and socio-demographic and clinical data from ~24,000 volunteers, aged 18-98 years, in ~7,000 family groups. Participants were recruited across Scotland, with some family members from further afield, from 2006-2011. Most (87%) participants were born in Scotland and 96% in the UK or Ireland. The cohort profile has been published^22^. GS:SFHS operates under appropriate ethical approvals, and all participants gave written informed consent. Generation Scotland is a collaboration between the University Medical Schools and National Health Service in Aberdeen, Dundee, Edinburgh and Glasgow (UK).

**GoDARTS (Genetics of Diabetes Audit and Research Tayside)** study recruits diabetic patients and non-diabetic matched controls in Tayside, Scotland; and details can be found elsewhere and at [diabetesgenetics.dundee.ac.uk](http://diabetesgenetics.dundee.ac.uk/)**.**

The KORA studies (Cooperative Health Research in the Region of Augsburg; German: Kooperative Gesundheitsforschung in der Region Augsburg) are a series of independent population based studies from the general population living in the region of Augsburg, Southern Germany^23^. **KORA F4** including 3,080 individuals was conducted from 2006-2008 as a follow-up study to KORA S4 (1999-2001).

The **Lothian Birth Cohort 1921 (LBC1921)** consists of 550 (234 male) relatively healthy individuals, assessed on cognitive and medical traits at a mean age of 79.1 years (SD = 0.6). They were born in 1921, most took part in the Scottish Mental Survey of 1932, and almost all lived independently in the Lothian region (Edinburgh City and surrounding area) of Scotland. A full description of participant recruitment and testing can be found elsewhere^24^. Genotyping was performed at the Wellcome Trust Clinical Research Facility, Edinburgh. Quality control measures were applied and 517 participants remained.

The **Lothian Birth Cohort 1936 (LBC1936)** consists of 1,091 relatively healthy individuals assessed on cognitive and medical traits at about 70 years of age. They were all born in 1936 and most took part in the Scottish Mental Survey of 1947. At baseline the sample of 548 men and 543 women had a mean age 69.6 years (s.d. = 0.8). They were all Caucasian, community-dwelling, and almost all lived in the Lothian region (Edinburgh city and surrounding area) of Scotland. A full description of participant recruitment and testing can be found elsewhere^24^. Genotyping was performed at the Wellcome Trust Clinical Research Facility, Edinburgh. Quality control measures were applied and 1,005 participants remained.

**LifeLines** is a multi-disciplinary prospective population-based cohort study examining in a unique three-generation design the health and health-related behaviours of 165,000 persons living in the North East region of The Netherlands. Study details can be found elsewhere^25^.

**LOLIPOP (London Life Sciences Prospective Population Study)** is a population based cohort study of ~30,000 South Asian and European white men and women, aged 35-75 years, recruited from the lists of 58 General Practitioners in West London, UK. Study details are given elsewhere^26^.

**LRGP (Leidsche Rijn GezondheidsProject)** cohort is a population-based cohort that includes over 10,000 residents of Leidsche Rijn (Utrecht, the Netherlands). Study details are given elsewhere^27^**.**

**OxBB (Oxford BioBank)** is a “collection of 30-50 year old healthy men and women living in Oxfordshire”. Study details can be found elsewhere^28^ and at [www.oxfordbiobank.org.uk](http://www.oxfordbiobank.org.uk/).

**SEARCH** (**Studies of Epidemiology and Risk factors in Cancer Heredity)** is a population-based study with cases ascertained through the Eastern Cancer Registration and Information Centre (<http://www.ecric.org.uk>). Study details can be found at [ccge.medschl.cam.ac.uk/search-study](http://ccge.medschl.cam.ac.uk/search-study/).

The **Study of Health in West Pomerania (SHIP)** is a cross-sectional, population based survey in a region in the Northeast of Germany. Study details are given elsewhere^29^.

**SIBS (Sisters in Breast Screening)** uses families identified through the breast screening program in the United Kingdom; and study details are given elsewhere^30^.

The **United Kingdom Household Longitudinal Study (UKHLS)**, also known as Understanding Society (https://www.understandingsociety.ac.uk) is a longitudinal panel survey of 40,000 UK households (England, Scotland, Wales and Northern Ireland) representative of the UK population. Participants are surveyed annually since 2009 and contribute information relating to their socioeconomic circumstances, attitudes, and behaviours via a computer assisted interview. The study includes phenotypical data for a representative sample of participants for a wide range of social and economic indicators as well as a biological sample collection encompassing biometric, physiological, biochemical, and haematological measurements and self-reported medical history and medication use. The United Kingdom Household Longitudinal Study has been approved by the University of Essex Ethics Committee and informed consent was obtained from every participant.

For a subset of individuals who took part in a nurse health assessment, blood samples were taken and genomic DNA extracted. Of these, 10,484 samples were genotyped at the Wellcome Trust Sanger Institute using the Illumina Infinium HumanCoreExome-12 v1.0BeadChip.

**Atherosclerosis Risk in Communities (ARIC),** is designed to look at risks and clinical outcomes associated with atherosclerosis in older population. To date, the study has collected information in approximately 4000 people aged 45-64 years old. Details can be on [www2.cscc.unc.edu/aric](https://www2.cscc.unc.edu/aric/).

**Collaborative Study on the Genetics of Alcoholism (COGA),** is a collaborative effort by the NIAAA to study the genetic effects on alcoholism. They have data on 2,255 extended families from six sites (SUNY Downstate Health Sciences Center, University of Connecticut, Indiana University, Washington University, University of Iowa, and The University of California at San Diego). Details can be on [www.niaaa.nih.gov/research/major-initiatives/collaborative-studies-genetics-alcoholism-coga-study](https://www.niaaa.nih.gov/research/major-initiatives/collaborative-studies-genetics-alcoholism-coga-study).

**Finnish Twin Cohort (FTC)** nation-wide population-based twin family study in Finland. It follows a series of cohort of twins in three stages stages, with twins born before 1958 (started in 1974), twins born 1975-1979 (started in 1991) and twins born 1983-1987 (starting from 1974, 1987 and started in 1994)1995.. Currently, there are 25,932 individuals in the study. Details can be found on [www.twinstudy.helsinki.fi](http://www.twinstudy.helsinki.fi) and reference^31^.

**Finland-United States Investigation of NIDDM Genetics (FUSION)** attempts to identify genetic risk for type 2 diabetes mellitus using a case-control sample. More study information can be found here: [fusion.sph.umich.edu/Pubs/papers/zeggini_diagram_t2dmeta_2008.pdf](https://fusion.sph.umich.edu/Pubs/papers/zeggini_diagram_t2dmeta_2008.pdf).

**Genetics and Epidemiology of Colorectal Cancer Consortium (GECCO)** studies colorectal cancer in a case-control study using data from over 40,000 participants.

**Genes for Good Facebook study (GFG)** is an application-based study started from the University of Michigan that uses Facebook as a platform to communicate with participants.

The **Health and Retirement Study (HRS)** is a longitudinal survey of a representative sample of Americans over the age of 50. The current sample is over 26,000 persons in 17,000 households. The study interviews respondents every two years about income and wealth, health and use of health services, work and retirement, and family connections. DNA was extracted from saliva collected during a face-to-face interview in the respondents' homes. These data represent respondents who provided DNA samples and signed consent forms in 2006, 2008, and 2010. Details can be found in reference^32^.

**ID1000** is a study in Netherlands where 1000 young adults participated in MRI studies at the Spinoza Center for Neuroimaging, in the Amsterdam Brain & Cognition research center.

**Multi-ethnic Cohort Study (MEC)** is an ethnically diverse cohort study based in Hawaii and California in the US that looks at the genetic risk that influences Cancer.

**METabolic Syndrome In Men (METSIM)** looks at risk of type 2 diabetes (T2D), cardiovascular disease (CVD), and insulin resistance in men aged from 45 to 73 years in eastern Finland.

**Montreal Heart Institute (MHI)** is a large hospital cohort based in Montreal studying cardiovascular diseases and its genetic risk factors.

**NAG-OZALC** is a study of alcohol disorders in a multi-cohort Australian twin-family sample. NIDA Nicotine Addiction Genetics [NAG] project is one of 3 coordinated studies that works with the OZALC data by identifying and working with the heavy smokers in the sample.

**Netherlands study of Cognition, Environment and Genes (NESCOG)** is a national representative sample of adults in Netherlands that investigates the underlying genetic factors related to intelligence.

**sardiNIA** is a study of longevity on a sample from Sardinia focused on the use of founder populations to simplify analysis of complex traits.

**TwinsUK:** A study of adult twins to study the genetic and environmental effects on age-related diseases and complex traits.

The **UK BiLEVE** samples comprised of 48,930 individuals selected for the UK Biobank Lung Exome Variant Evaluation (UK BiLEVE) project^12^. **UK Biobank** (http://www.ukbiobank.ac.uk/) contains data from 502,682 individuals including UK BiLEVE (94% of self-reported European ancestry) with extensive health and lifestyle questionnaire data, physical measures and DNA^33^.

**Women’s Health Initiative (WHI)** is a complex study that is designed with clinical trials and observational cohorts in order to look at the risk factors in aging women.

**The Copenhagen Ischaemic Heart Disease Study (CIHDS)** study is comprised of cases with myocardial infarction and other major acute coronary syndromes. The cases were recruited from Copenhagen University Hospital during the period from 1991 to 2009. In addition to a diagnosis of acute coronary syndrome, these cases also had stenosis or atherosclerosis on coronary angiography and/or positive results on exercise electrocardiography. Cases were classified by World Health Organization International Classification of Diseases-Eighth Revision, codes 410 to 414; International Classification of Diseases-Tenth Revision, codes I20 to I25, and through review of all hospital admissions and diagnoses entered in the national Danish Patient Registry and all causes of death entered in the national Danish Causes of Death Registry, as previously described^34^.

**The Copenhagen General Population Study (CGPS)** is a population-based prospective study initiated in 2003 with ongoing enrolment^34^. Participants were selected on the basis of the national Danish Civil Registration System to reflect the adult Danish population age 20 to ≥80 years. Data were obtained from a questionnaire, a physical examination, and blood samples including deoxyribonucleic acid extraction. Follow-up was 100% complete; that is, no participant was lost to follow-up.

**Copenhagen City Heart Study (CCHS)** is a population-based prospective study initiated in 1976 with follow-up examinations from 1981 to 1983, 1991 to 1994, and 2001 to 2003^35^. Selection of individuals for the CCHS was based on the same criteria as for the CGPS. Information on diagnosis of CAD (defined as WHO ICD 8 410 to 414 and WHO-ICD 10 I20 to I25) was collected and veriﬁed from 1976 until 2010 by reviewing all hospital admissions and diagnoses entered in the national Danish Patient Registry, and by reviewing all causes of death entered in the national Danish Causes of Death Registry^35, 36^. Again, follow-up was 100% complete for both non-fatal coronary outcomes and mortality.

**European Investigation into Cancer and Nutrition-CVD (EPIC-CVD):** EPIC is a multi-centre prospective cohort study^37^ of 519,978 participants (366,521 women and 153,457 men, mostly aged 35–70 years) recruited between 1992 and 2000 in 23 centres located in 10 European countries. Participants were invited mainly from population-based registers (Denmark, Germany, certain Italian centres, the Netherlands, Norway, Sweden, UK)^38^. Other sampling frameworks included: blood donors (Spain and Turin and Ragusa in Italy); screening clinic attendees (Florence in Italy and Utrecht in the Netherlands); people in health insurance programmes (France); and health conscious individuals (Oxford, UK)^38^. About 97% of the participants were of white European ancestry. Prevalent CAD was ascertained through self-reported history of MI or angina, or registry-ascertained CAD event prior to baseline. EPIC-CVD employs a nested case-cohort design, analogous to the EPIC-InterAct study for type-2 diabetes^39^ which established a common set of referents through selection of a random sample of the entire cohort (“subcohort”). Incident CAD cases have been defined as fatal and non-fatal MI and other major acute coronary events, according to ICD-10 codes I20-I25. All centres have recorded cause-specific mortality through mortality registries and/or active follow-up, and have ascertained and validated incident fatal and non-fatal CAD through a combination of methods (eg, morbidity registers, general practice records, MONICA registries, self-report, clinical records^39^).

**Bangladesh Risk of Acute Vascular Events (BRAVE)** is a retrospective case-control study of first-ever confirmed acute myocardial infarction (MI) in Bangladesh. Patients (male or female; age between 30-80 years) admitted to the emergency rooms of the collaborating hospital in Dhaka, Bangladesh were eligible for inclusion as MI cases if they fulfilled all of the following criteria: i) presented within 24 hours of the onset of sustained clinical symptoms suggestive of MI lasting longer than 20 minutes, including chest pain and breathlessness; ii) had ECG changes indicative of MI (new pathologic Q waves, at least 1 mm ST elevation in any 2 or more contiguous limb leads or a new left bundle branch block, or new persistent ST-T wave changes diagnostic of a non-Q wave MI) with a subsequent confirmation by troponin-I measurements; and iii) had no previous cardiovascular diseases; defined as self-reported history of angina, MI, coronary revascularisation, transient ischaemic attack, stroke or evidence of CAD on prior ECG or in other medical records. Participants were not recruited into BRAVE if any of the following features had been evident: i) a previous history of cardiovascular disease (including self-reported MI, angina, coronary revascularization, stroke, transient ischaemic attack, or peripheral vascular disease, and, in cases, presence of cardiogenic shock); ii) a history of a viral or bacterial infection in the previous 2 weeks; iii) current hospitalization for acute cerebrovascular events; iv) MI secondary to any surgery; v) documented chronic conditions, such as malignancy, any chronic infection, leprosy, malaria or other bacterial/parasitic infections, chronic inflammatory disorders, hepatitis or renal failure on past medical history; vi) pregnancy or related conditions; or vii) unable to provide consent. Controls were hospital based and frequency-matched to cases on age (within 5 year age bands) and sex, and without a self-reported history of cardiovascular disease.

**Pakistan Risk of Myocardial Infarction Study (PROMIS)** is an ongoing retrospective case-control study of first-ever confirmed acute MI in Pakistan. Since 2005, the study has enrolled close to 18,500 MI cases and equivalent number of controls; the present investigation has included all MI cases and controls that had been enrolled until 2011. Patients aged 30-80 years who were admitted to the emergency rooms of nine recruitment centres across Pakistan^40^ were eligible for inclusion as cases if they fulfilled all of the following criteria: symptoms within 24 hours of hospital presentation; typical ECG changes; and positive troponin-I test. To identify referents from approximately the same source population as the cases, controls were identified contemporaneously in the same hospitals as the index cases and selected from among people who had no history of CVD and who were: visitors of patients attending the outpatient department; patients attending outpatient departments for routine non-cardiac complaints; or non-blood relatives visiting index MI cases. Controls were frequency-matched to MI cases by sex and age (5-year bands). People with recent illnesses or infections were not eligible.

MONICA Risk Genetics, Archiving and Monograph (**MORGAM**) is a consortium of cohort studies on cardiovascular diseases, whose data have been harmonized into one database for joint analysis^41^. For the current analysis, the following cohorts were included: Brianza cohorts 01, 02 and 03 (Italy); the placebo cohort of the ATBC Study (Finland); FINRISK cohort 1992 and 1997 (Finland); Lille, Strasbourg and Toulouse cohorts of the PRIME study (France); Augsburg (KORA) cohorts S1, S2 and S3 (Germany); and Belfast cohort of the PRIME study (Northern Ireland) . The cohorts were based on random population samples, except ATBC which included only smokers, and they were recruited between years 1984 and 1997. For genetic analyses, a case-cohort design was used.

The **INTERVAL** study comprised about 50,000 participants nested within a randomised trial of varying blood donation intervals46. Between mid-2012 and mid-2014, whole-blood donors aged 18 years and older were consented and recruited at 25 centers of England’s National Health Service Blood and Transplant (NHSBT). Participants completed an online questionnaire including questions about demographic characteristics (e.g., age, sex, ethnic group), anthropometry (height, weight), lifestyle (e.g., alcohol and tobacco consumption) and diet. Participants were generally in good health because blood donation criteria exclude people with a history of major diseases (such as myocardial infarction, stroke, cancer, HIV, and hepatitis B or C) and those who have had recent illness or infection

## Study-level Quality Control Procedures

**Consortium for the Genetics of Smoking Behaviour (CGSB)**

For AIRWAVE, ASCOT, 1958BC, BRIGHT, DIABNORD, EFSOCH, EGCUT, EMBRACE, FENLAND, FIA3, GLACIER, GoDARTS, KORA F4, LifeLines, LOLIPOP, LRGP, OXBB, SEARCH, SHIP, SIBS, genotype calling and quality control were carried out in accordance with the Exome-chip Quality Control SOP Version 5 (20/11/2012), as developed within the UK exome-chip consortium (by Mahajan, A., Robertson, N. and Rayner, W). Genotypes were initially called using Gencall in Illumina’s Genome Studio software (Illumina Inc. Illumina GenCall Data Analysis Software, 2005). Quality control of SNPs and samples was subsequently performed at study level. Initial filters applied excluded SNPs with very low call rate (<90%) and samples with low call rate, heterozygosity outliers, duplicates, gender mismatches and ancestral outliers. SNPs with missing data were then recalled using genotype calling software zCall^42^. All alleles were mapped to the forward strand of human genome build 37 and secondary exclusions were applied to remove SNPs with low call rate (<99%) or deviations from Hardy Weinberg Equilibrium (*P<*10^-4^). Samples with call rate <99% and heterozygosity outliers were also excluded.

For GS:SFHS, CROATIA-Korcula and LBC1936, LBC1921 , genotypes were called using Gencall in Illumina’s Genome Studio software (<https://www.illumina.com/Documents/products/technotes/technote_gencall_data_analysis_software.pdf>) via the CHARGE Consortium joint calling cluster file (<http://www.chargeconsortium.com/main/exomechip>) and quality control of the genotype data was undertaken according to the CHARGE exome chip best practices, described elsewhere^43^.

UKHLS: Genotype calling was performed using the Illumina GenCall software. Sample-level quality control (QC) was performed using the following filters: call rate <98%, autosomal heterozygosity outliers (>3 SD), gender mismatches, duplicates as established by identity by descent (IBD) analysis (PI_HAT >0.9), ethnic outliers as determined by combining with 1000 Genomes Project data and carrying out IBD followed by multidimensional scaling. In total, 9,965 samples passed QC. Variant-level QC was performed as follows: variants were mapped to forward strand of human genome build 37. Variants with Hardy-Weinberg equilibrium P < 1×10^-4^, a call rate < 98% and poor genotype clustering values (< 0.4) were removed, as well as Y-chromosome and mitochondrial variants.

**GSCAN**

Study-level QC procedures and analysis plan for the GSCAN participating cohorts can be found at: <http://gscan.sph.umich.edu/exome/analysis_plan>.

**INTERVAL**

The genotyping protocol and QC for the INTERVAL samples (n~50,000) have been described previously in detail^44^. Briefly, DNA extracted from buffy coat was used to assay approximately 830,000 variants on the Affymetrix Axiom UK Biobank genotyping array at Affymetrix (Santa Clara, California, US). Genotyping was performed in multiple batches of approximately 4,800 samples each. Sample QC was performed including exclusions for sex mismatches, low call rates, duplicate samples, extreme heterozygosity and non-European descent. An additional exclusion made for this study was of one participant from each pair of close (first- or second-degree) relatives, defined as $\hat{\pi}$>0.187. Identity-by-descent was estimated using a subset of variants with a call rate >99% and MAF >0.05 in the merged dataset of both subcohorts, pruned for linkage disequilibrium (LD) using PLINK v1.9. Multi-dimensional scaling was performed using PLINK v1.9 to create components to account for ancestry in genetic analyses.

Prior to imputation, additional variant filtering steps were performed to establish a high-quality imputation scaffold. In summary, 654,966 high quality variants (autosomal, non-monomorphic, bi-allelic variants with Hardy Weinberg Equilibrium (HWE) *P*>5x10^-6^, with a call rate of >99% across the INTERVAL genotyping batches in which a variant passed QC, and a global call rate of >75% across all INTERVAL genotyping batches) were used for imputation. Variants were phased using SHAPEIT3 and imputed using a combined 1000 Genomes Phase 3-UK10K reference panel. Imputation was performed via the Sanger Imputation Server (<https://imputation.sanger.ac.uk>) resulting in 87,696,888 imputed variants.

**CHD Exome+ Consortium**

The CHD Exome + consortium is composed of 8 different cohorts, 6 from Europe (EPIC-CVD, CCHS, CGPS, CIHDS, PROSPER, MORGAM) and 2 from South Asia (BRAVE, PROMIS). The three Copenhagen collections (CCHS, CIHDS, CGPS) were genotyped in Copenhagen, all other genotyping was performed in Cambridge, UK. Two versions of the Exome+ chip were used (both with the same standard Exome chip content but different custom content) necessitating some collections to be genotyped in batches (CIHDS, CGPS, PROMIS, BRAVE). Consequently, genotype calling was done at the batch level, with all batches going through the same calling and QC pipeline in Cambridge. EPIC-CVD and CCHS were only genotyped on version 1 of the chip, while PROSPER, were only genotyped on version 2 of the chip and hence were genotyped as single batches. Details of the consortium design are summarised in the table below.

| **Ethnicity** | **Collection** | **Study design** | **Number of genotyping batches** | **Association Study** |
| --- | --- | --- | --- | --- |
| South Asian | BRAVE | Case-control | 2 | BRAVE |
|  | PROMIS | Case-control | 3* | PROMIS |
| European | EPIC-CVD | Case-cohort | 1 | EPIC |
|  | CCHS | Prospective | 1 | CCHS |
|  | CGPS | Cross-sectional | 2 | CGPS |
|  | CIHDS | Case series | 2 | CIHDS |
|  | MORGAM | Case-cohort | 1 | MORGAM |
|  | PROSPER | Nested case-control within trial | 1 | PROSPER |
| **Total number** | **8** | **-** | **13** | **8** |

* Note, PROMIS was genotyped on ‘version 1’ of the chip and in two batches on ‘version 2’ of the chip, as samples were still being recruited while genotyping was being undertaken.

QC steps were undertaken at both the batch and study level as follows:

Genotype batch-level QC:

- Sample exclusions based on pre-genotype calling
  - raw intensities pre-calling (poor performing plates/arrays/sample intensity outliers)
- Sample exclusions post genotype calling
  - heterozygosity (samples +/-3SD from batch mean heterozygosity)
  - call rate (samples more than 3SD less than batch mean, equates to ~0.97)
  - sex mismatches or genotype discordance with previous arrays
- SNV exclusions based on:
  - call rate (SNVs with call rate <0.97 in CHD cases or controls)
  - HWE (Z^2^ > 24 [equivalent to *P*<1x10^-06^] for common SNVs [MAF ≥ 5%], Z^2^ > 64 [equivalent to *P*<1x10^-15^] for rare SNVs [MAF<5%] in controls or all samples in genotyping batch)
  - Variants failing visual cluster plot inspections.

Study-level QC:

- Sample exclusions based on:
  - Ancestry outliers from PCA
  - Duplicates identified from kinship
- SNV exclusions based on:
  - HWE in controls or all samples in study (Z^2^ > 24 [equivalent to *P*<1x10^-06^ ] for common SNVs [MAF ≥ 5%], Z^2^ > 64 [equivalent to *P*<1x10^-15^ ] for rare SNVs [MAF<5%])

## UK Biobank Phenotype Information

Cigarettes per day (CPD): We defined CPD using the combination of phenotype codes of 2887 (number of cigarettes previously smoked daily), 3456 (number of cigarettes currently smoked daily), and 6183 (number of cigarettes previously smoked daily (current cigar/pipe smokers)). Extreme outliers with values >60 were removed. The phenotype was binned and recoded according to 1-10-> 1, 11-20-> 2, 21-30-> 3, >30-> 4.

Smoking Initiation: We coded the ever-regular cigarette smoker as 2 and the individuals that were never a regular cigarette smoker as 1.

We defined an individual as ever-regular smokers if:

1. They answered the field 2644 (light smokers, at least 100 smokes in lifetime) as “Yes”; or
2. They responded "Hand-rolled cigarettes" or "Manufactured cigarettes" to 2877 (type of tobacco previously smoked); or
3. They were former cigarette smokers but currently use a different tobacco product, as indicated by a non-null response to 6183; or
4. They responded "Hand-rolled cigarettes" or "Manufactured cigarettes" to 3446 (Type of tobacco currently smoked).

The individuals that were deemed a never regular smoker if:

1. They answered “No” to 2644; or
2. They responded "I have never smoked" to 1249 (past tobacco smoking).

Pack-Years: For current smokers, the number of years of smoking was defined as difference between 21003 (age when attended assessment centre) and 3436 (age started smoking in current smokers). For previous smokers, the number of years of smoking was defined by the difference between 2897 (age stopped smoking) and 2867 (age started smoking in former smokers). The number of years of smoking that was less than one (1) was set to missing. Pack-years was then calculated as the non-binned CPD, divided by 20, times the number of years of smoking. The numbers were log transformed to reduce the impact of potential outliers.

Smoking Cessation: we coded current smoker as ‘2’, and former smoker as ‘1’. Specifically, we defined an individual as a former smoker if:

1. They answered yes to 2644; or
2. They responded "Hand-rolled cigarettes" or "Manufactured cigarettes" to 2877.

We define an individual to be a current smoker if they answered "Hand-rolled cigarettes" or "Manufactured cigarettes" to 3446.

## Phenotypic Variance Explained

We estimated the proportion of variance explained by the set of all conditionally independently associated variants (**Tables 1-3** and **Suppl. Table 3**). The joint effects of variants in a locus were approximated by ${\hat{\vec{\beta}}}_{JOINT}=\mathbf{V}_{\mathbf{META}}^{\mathbf{-1}}\vec{U}_{META}$, where $\vec{U}_{META}$ is the single variant score statistics and $\mathbf{V}_{\mathbf{META}}$ is the covariance matrix between them. The phenotypic variance explained by the independently associated variants in a locus is given by ${\hat{\vec{\beta}}}_{joint}^{T}\mathrm{cov}\left( G \right){\hat{\vec{\beta}}}_{JOINT}$, where cov(G) is the partial covariance between different variants as estimated from $\mathbf{V}_{\mathbf{META}}$. Together the phenotypic variance explained by the novel variants were 0.53% (SI), 0.0026% (PY), 0.72% (CPD) and 0.016% (SC). The phenotypic variance explained by both novel and known variants were 0.61% (SI), 0.31% (PY), 1.2% (CPD), and 0.027% (SC). Our novel variants substantially improved the phenotypic variance explained, yet the total phenotypic variance explained remained low for smoking related traits.

## Genes of interest

Interestingly, some of the associated variants appear to have regulatory roles on nicotine addiction related genes. For example, rs11776293 (an intronic variant in *EPHX2*; **Table 2**), was an eQTL for *CHRNA2*, with the T allele increasing the gene’s expression in brain cerebellum in GTEx (*P*=2.5x10^-5^; β=0.61). *CHRNA2*, a gene that showed nominal association with pack-years in our gene-based tests (**Suppl.** **Table 6**), encodes the α2 subunit nicotinic acetylcholine receptor gene. *CHRNA2* has previously been reported with nominal evidence of association with common SNVs in small candidate gene studies^45, 46^. We also identified an association of *CHRNA2* with pack-years in the gene-based tests, although this was mostly driven by a single variant, rs56229264. Common variants at this locus have been shown to be associated with lung cancer and cannabis use disorder^47^, and potentially regulating the expression of *CHRNA2* in the cerebellum.^48^

## References

1. Yeager M, Orr N, Hayes RB, Jacobs KB, Kraft P, Wacholder S *et al.* Genome-wide association study of prostate cancer identifies a second risk locus at 8q24. *Nature genetics* 2007; **39**(5)**:** 645-649.

2. Amundadottir L, Kraft P, Stolzenberg-Solomon RZ, Fuchs CS, Petersen GM, Arslan AA *et al.* Genome-wide association study identifies variants in the ABO locus associated with susceptibility to pancreatic cancer. *Nature genetics* 2009; **41**(9)**:** 986-990.

3. Petersen GM, Amundadottir L, Fuchs CS, Kraft P, Stolzenberg-Solomon RZ, Jacobs KB *et al.* A genome-wide association study identifies pancreatic cancer susceptibility loci on chromosomes 13q22.1, 1q32.1 and 5p15.33. *Nature genetics* 2010; **42**(3)**:** 224-228.

4. Landi MT, Chatterjee N, Yu K, Goldin LR, Goldstein AM, Rotunno M *et al.* A genome-wide association study of lung cancer identifies a region of chromosome 5p15 associated with risk for adenocarcinoma. *American journal of human genetics* 2009; **85**(5)**:** 679-691.

5. Moore C, Sambrook J, Walker M, Tolkien Z, Kaptoge S, Allen D *et al.* The INTERVAL trial to determine whether intervals between blood donations can be safely and acceptably decreased to optimise blood supply: study protocol for a randomised controlled trial. *Trials* 2014; **15:** 363.

6. Tobacco and Genetics Consortium. Genome-wide meta-analyses identify multiple loci associated with smoking behavior. *Nature genetics* 2010; **42**(5)**:** 441-447.

7. Thorgeirsson TE, Gudbjartsson DF, Surakka I, Vink JM, Amin N, Geller F *et al.* Sequence variants at CHRNB3-CHRNA6 and CYP2A6 affect smoking behavior. *Nature genetics* 2010; **42**(5)**:** 448-453.

8. Thakur GA, Sengupta SM, Grizenko N, Choudhry Z, Joober R. Family-based association study of ADHD and genes increasing the risk for smoking behaviours. *Archives of disease in childhood* 2012; **97**(12)**:** 1027.

9. Bloom AJ, Baker TB, Chen L-S, Breslau N, Hatsukami D, Bierut LJ *et al.* Variants in two adjacent genes, EGLN2 and CYP2A6, influence smoking behavior related to disease risk via different mechanisms. *Human Molecular Genetics* 2014; **23**(2)**:** 555-561.

10. Timofeeva MN, McKay JD, Smith GD, Johansson M, Byrnes GB, Chabrier A *et al.* Genetic polymorphisms in 15q25 and 19q13 loci, cotinine levels, and risk of lung cancer in EPIC. *Cancer Epidemiol Biomarkers Prev* 2011; **20**(10)**:** 2250-2261.

11. Siedlinski M, Cho MH, Bakke P, Gulsvik A, Lomas DA, Anderson W *et al.* Genome-wide association study of smoking behaviours in patients with COPD. *Thorax* 2011; **66**(10)**:** 894-902.

12. Wain LV, Shrine N, Miller S, Jackson VE, Ntalla I, Soler Artigas M *et al.* Novel insights into the genetics of smoking behaviour, lung function, and chronic obstructive pulmonary disease (UK BiLEVE): a genetic association study in UK Biobank. *Lancet Respir Med* 2015; **3**(10)**:** 769-781.

13. Hancock DB, Reginsson GW, Gaddis NC, Chen X, Saccone NL, Lutz SM *et al.* Genome-wide meta-analysis reveals common splice site acceptor variant in CHRNA4 associated with nicotine dependence. *Transl Psychiatry* 2015; **5:** e651.

14. Elliott P, Vergnaud A-C, Singh D, Neasham D, Spear J, Heard A. The Airwave Health Monitoring Study of police officers and staff in Great Britain: Rationale, design and methods. *Environmental Research* 2014; **134:** 280-285.

15. Sever PS, Dahlof B, Poulter NR, Wedel H, Beevers G, Caulfield M *et al.* Rationale, design, methods and baseline demography of participants of the Anglo-Scandinavian Cardiac Outcomes Trial. ASCOT investigators. *J Hypertens* 2001; **19**(6)**:** 1139-1147.

16. Strachan DP, Rudnicka AR, Power C, Shepherd P, Fuller E, Davis A *et al.* Lifecourse influences on health among British adults: Effects of region of residence in childhood and adulthood. *International journal of epidemiology* 2007; **36**(3)**:** 522-531.

17. Caulfield M, Munroe P, Pembroke J, Samani N, Dominiczak A, Brown M *et al.* Genome-wide mapping of human loci for essential hypertension. *Lancet* 2003; **361**(9375)**:** 2118-2123.

18. Zemunik T, Boban M, Lauc G, Jankovic S, Rotim K, Vatavuk Z *et al.* Genome-wide association study of biochemical traits in Korcula Island, Croatia. *Croatian medical journal* 2009; **50**(1)**:** 23-33.

19. Hallmans G, Agren A, Johansson G, Johansson A, Stegmayr B, Jansson JH *et al.* Cardiovascular disease and diabetes in the Northern Sweden Health and Disease Study Cohort - evaluation of risk factors and their interactions. *Scand J Public Health Suppl* 2003; **61:** 18-24.

20. Knight B, Shields BM, Hattersley AT. The Exeter Family Study of Childhood Health (EFSOCH): study protocol and methodology. *Paediatr Perinat Epidemiol* 2006; **20**(2)**:** 172-179.

21. Nelis M, Esko T, Magi R, Zimprich F, Zimprich A, Toncheva D *et al.* Genetic structure of Europeans: a view from the North-East. *PloS one* 2009; **4**(5)**:** e5472.

22. Smith BH, Campbell A, Linksted P, Fitzpatrick B, Jackson C, Kerr SM *et al.* Cohort Profile: Generation Scotland: Scottish Family Health Study (GS:SFHS). The study, its participants and their potential for genetic research on health and illness. *International journal of epidemiology* 2013; **42**(3)**:** 689-700.

23. Holle R, Happich M, Lowel H, Wichmann HE, Group MKS. KORA--a research platform for population based health research. *Gesundheitswesen* 2005; **67 Suppl 1:** S19-25.

24. Taylor AM, Pattie A, Deary IJ. Cohort Profile Update: The Lothian Birth Cohorts of 1921 and 1936. *International journal of epidemiology* 2018.

25. Stolk RP, Rosmalen JG, Postma DS, de Boer RA, Navis G, Slaets JP *et al.* Universal risk factors for multifactorial diseases: LifeLines: a three-generation population-based study. *Eur J Epidemiol* 2008; **23**(1)**:** 67-74.

26. Chambers JC, Zhang W, Lord GM, van der Harst P, Lawlor DA, Sehmi JS *et al.* Genetic loci influencing kidney function and chronic kidney disease. *Nature genetics* 2010; **42**(5)**:** 373-375.

27. Grobbee DE, Hoes AW, Verheij TJ, Schrijvers AJ, van Ameijden EJ, Numans ME. The Utrecht Health Project: optimization of routine healthcare data for research. *Eur J Epidemiol* 2005; **20**(3)**:** 285-287.

28. Tan GD, Neville MJ, Liverani E, Humphreys SM, Currie JM, Dennis L *et al.* The in vivo effects of the Pro12Ala PPARγ2 polymorphism on adipose tissue NEFA metabolism: the first use of the Oxford Biobank. *Diabetologia* 2006; **49**(1)**:** 158-168.

29. John U, Greiner B, Hensel E, Ludemann J, Piek M, Sauer S *et al.* Study of Health In Pomerania (SHIP): a health examination survey in an east German region: objectives and design. *Soz Praventivmed* 2001; **46**(3)**:** 186-194.

30. Kataoka M, Antoniou A, Warren R, Leyland J, Brown J, Audley T *et al.* Genetic models for the familial aggregation of mammographic breast density. *Cancer Epidemiol Biomarkers Prev* 2009; **18**(4)**:** 1277-1284.

31. Kaprio J. The Finnish Twin Cohort Study: an update. *Twin research and human genetics : the official journal of the International Society for Twin Studies* 2013; **16**(1)**:** 157-162.

32. Sonnega A, Faul JD, Ofstedal MB, Langa KM, Phillips JW, Weir DR. Cohort Profile: the Health and Retirement Study (HRS). *International journal of epidemiology* 2014; **43**(2)**:** 576-585.

33. Bycroft C, Freeman C, Petkova D, Band G, Elliott LT, Sharp K *et al.* Genome-wide genetic data on ~500,000 UK Biobank participants. *bioRxiv* 2017.

34. Nordestgaard BG, Benn M, Schnohr P, Tybjaerg-Hansen A. Nonfasting triglycerides and risk of myocardial infarction, ischemic heart disease, and death in men and women. *JAMA* 2007; **298**(3)**:** 299-308.

35. Kamstrup PR, Tybjaerg-Hansen A, Steffensen R, Nordestgaard BG. Genetically elevated lipoprotein(a) and increased risk of myocardial infarction. *JAMA* 2009; **301**(22)**:** 2331-2339.

36. Varbo A, Benn M, Tybjaerg-Hansen A, Jorgensen AB, Frikke-Schmidt R, Nordestgaard BG. Remnant cholesterol as a causal risk factor for ischemic heart disease. *J Am Coll Cardiol* 2013; **61**(4)**:** 427-436.

37. Riboli E, Hunt KJ, Slimani N, Ferrari P, Norat T, Fahey M *et al.* European Prospective Investigation into Cancer and Nutrition (EPIC): study populations and data collection. *Public Health Nutr* 2002; **5**(6B)**:** 1113-1124.

38. Danesh J, Saracci R, Berglund G, Feskens E, Overvad K, Panico S *et al.* EPIC-Heart: the cardiovascular component of a prospective study of nutritional, lifestyle and biological factors in 520,000 middle-aged participants from 10 European countries. *Eur J Epidemiol* 2007; **22**(2)**:** 129-141.

39. Langenberg C, Sharp S, Forouhi NG, Franks PW, Schulze MB, Kerrison N *et al.* Design and cohort description of the InterAct Project: an examination of the interaction of genetic and lifestyle factors on the incidence of type 2 diabetes in the EPIC Study. *Diabetologia* 2011; **54**(9)**:** 2272-2282.

40. Saleheen D, Zaidi M, Rasheed A, Ahmad U, Hakeem A, Murtaza M *et al.* The Pakistan Risk of Myocardial Infarction Study: a resource for the study of genetic, lifestyle and other determinants of myocardial infarction in South Asia. *Eur J Epidemiol* 2009; **24**(6)**:** 329-338.

41. Evans A, Salomaa V, Kulathinal S, Asplund K, Cambien F, Ferrario M *et al.* MORGAM (an international pooling of cardiovascular cohorts). *International journal of epidemiology* 2005; **34**(1)**:** 21-27.

42. Goldstein JI, Crenshaw A, Carey J, Grant GB, Maguire J, Fromer M *et al.* zCall: a rare variant caller for array-based genotyping: genetics and population analysis. *Bioinformatics* 2012; **28**(19)**:** 2543-2545.

43. Grove ML, Yu B, Cochran BJ, Haritunians T, Bis JC, Taylor KD *et al.* Best practices and joint calling of the HumanExome BeadChip: the CHARGE Consortium. *PloS one* 2013; **8**(7)**:** e68095.

44. Astle WJ, Elding H, Jiang T, Allen D, Ruklisa D, Mann AL *et al.* The Allelic Landscape of Human Blood Cell Trait Variation and Links to Common Complex Disease. *Cell* 2016; **167**(5)**:** 1415-1429 e1419.

45. Yang J, Wang S, Yang Z, Hodgkinson CA, Iarikova P, Ma JZ *et al.* The contribution of rare and common variants in 30 genes to risk nicotine dependence. *Mol Psychiatry* 2015; **20**(11)**:** 1467-1478.

46. Wang S, A DvdV, Xu Q, Seneviratne C, Pomerleau OF, Pomerleau CS *et al.* Significant associations of CHRNA2 and CHRNA6 with nicotine dependence in European American and African American populations. *Human genetics* 2014; **133**(5)**:** 575-586.

47. Demontis D, Rajagopal VM, Als TD, Grove J, Pallesen J, Hjorthoj C *et al.* Genome-wide association study implicates *CHRNA2* in cannabis use disorder. *bioRxiv* 2018.

48. McKay JD, Hung RJ, Han Y, Zong X, Carreras-Torres R, Christiani DC *et al.* Large-scale association analysis identifies new lung cancer susceptibility loci and heterogeneity in genetic susceptibility across histological subtypes. *Nature genetics* 2017; **49**(7)**:** 1126-1132.
